# Knockout of the sulfide: quinone oxidoreductase SQR reduces growth of HCT116 tumor xenograft

**DOI:** 10.1016/j.redox.2025.103650

**Published:** 2025-04-24

**Authors:** Ting Lu, Qingda Wang, Yuping Xin, Xiaohua Wu, Yang Wang, Yongzhen Xia, Luying Xun, Huaiwei Liu

**Affiliations:** a School of Health and Life Sciences, University of Health and Rehabilitation Sciences Qingdao Hospital (Qingdao Municipal Hospital), University of Health and Rehabilitation Sciences, Qingdao, 266071, People's Republic of China; b State Key Laboratory of Microbial Technology, Shandong University, Qingdao, 266200, People's Republic of China; c Origin Biotechnology Private Limited, 2 Venture Drive, 608526, Singapore; d School of Molecular Biosciences, Washington State University, Pullman, WA, 991647520, USA

**Keywords:** Colorectal cancer, HCT116, SQR, Polysulfides, Tumor xenograft, ALDOA

## Abstract

Colorectal cancer (CRC) exhibits significant diversity and heterogeneity, posing a requirement for novel therapeutic targets. Polysulfides are associated with CRC progression and immune evasion, but the underlying mechanisms are not fully understood. Sulfide: quinone oxidoreductase (SQR), a mitochondrial flavoprotein, catalyzes hydrogen sulfide (H_2_S) oxidation and polysulfides production. Herein, we explored its role in CRC pathogenesis and its potential as a therapeutic target. Our findings revealed that SQR knockout disrupted polysulfides homeostasis, diminished mitochondrial function, impaired cell proliferation, and triggered early apoptosis in HCT116 CRC cells. Moreover, the SQR knockout led to markedly reduced tumor sizes in mice models of colon xenografts. Although the transcription of glycolytic genes remained largely unchanged, metabolomic analysis demonstrated a reprogramming of glycolysis at the fructose-1,6-bisphosphate degradation step, catalyzed by aldolase A (ALDOA). Both Western blot analysis and enzymatic assays confirmed the decrease in ALDOA levels and activity. In conclusion, the study establishes the critical role of SQR in mitochondrial function and metabolic regulation in CRC, with its knockout leading to metabolic reprogramming and diminished tumor growth in HCT116 tumor xenografts. These insights lay a foundation for the development of SQR-targeted therapies for CRC.

## Highlights

• The mitochondrial flavoprotein SQR plays a pivotal role in maintaining mitochondrial health.
• Knocking out SQR leads to glycolysis reprogramming in HCT116 tumor xenograft.
• Knocking out SQR reduces size of the HCT116 tumor xenograft.

## Graphical abstract

**Figure:**
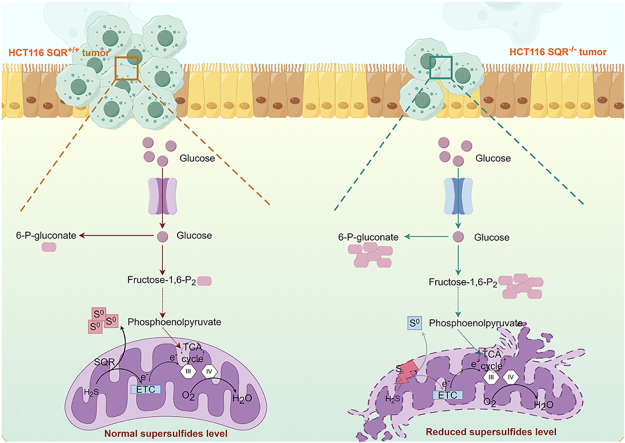

## 1 Introduction

An increasing body of research has established hydrogen sulfide (H_2_S) as a crucial regulator in a diverse array of physiological and pathological processes within mammalian cells. These processes encompass mitochondrial energy metabolism, embryonic development, cardiac function, tumorigenesis, innate immunity, and antiviral defense mechanisms [[1], [2], [3], [4]]. H_2_S exerts its signaling roles through three main mechanisms: first, by engaging in multiple reactions with various reactive oxygen species (ROS) and reactive nitrogen species (RNS); second, by interacting with metalloproteins or the metal centers within proteins; and third, by post-translationally modifying cysteine residues in proteins, leading to protein persulfidation (or sulfhydration) [[5], [6], [7], [8]].

Polysulfides including hydrogen polysulfide (HS_n_H, n ≥ 2), glutathione polysulfide (GS_n_H, n ≥ 2), cysteine polysulfide (Cys-S_n_H, n ≥ 2), and so on. These chemicals are collectively referred to as sulfane sulfur (S^0^), supersulfides or reactive sulfur species (RSS) in recent literatures [[9], [10], [11]]. In mammalian cells, polysulfides are mainly generated in two organelles. Cystathionine β-synthase (CBS), cystathionine γ-lyase (CSE), and 3-mercaptopyruvate sulfurtransferase (MST) are located in cytoplasm. The former two generate persulfidated and polysulfidated l-cysteine (Cys-S_n_H compounds) by degrading cystine, while the later produces HS_n_H via degrading 3-mercaptopyruvate [[12], [13], [14], [15]]. Cysteinyl-tRNA synthetase (CARS2) and sulfide:quinone oxidoreductase (SQR) are located in mitochondria. The former generates Cys-SSH from cysteine, while the latter oxidizes H_2_S to polysulfides. Among these enzymes, only SQR is the specific polysulfides-producing enzyme [[16], [17], [18], [19]]. As to others, producing polysulfides is more like a side-reaction as their primary activities are for basic cell processes (e.g., cysteine synthesis and protein translation).

CRC is the second leading cause of cancer-related deaths globally, and its intrinsic and acquired resistance to drugs highlights the requirement for novel therapeutic targets [20,21]. In recent years, increasing evidences have emerged and indicated that polysulfides are linked with CRC development [[22], [23], [24], [25]]. For instance, human mucosa tissue displays relatively higher CBS expression level compared to other tissues, and CBS knockdown results in decreased growth of colon tumor xenograft and reduced neovessel density [22]. Similarly, CSE knockdown or activity inhibition decreases the SW480 cell proliferation, migration, and tumor xenograft growth [23]. The expression of persulfide dioxygenase ETHE1 is elevated in colonic adenocarcinoma, with the extent of increase correlating with CRC tumor grade [26]. Given its role as the sole specific polysulfides-producing enzyme, SQR is hypothesized to play a significant role in CRC pathogenesis, although no studies have yet explored this relationship.

Previously, we found that SQR knockout resulted in abnormal mitochondria morphology and physiology in the eukaryotic model microorganism *Schizosaccharomyces pombe* [27]. Considering the central role of mitochondria as the powerhouse of the mammalian cell, we proposed that SQR knockout may affect CRC cell proliferation and tumor development. In this study, we constructed HCT116 SQR knockout cell line (*sqr*^−/−^) and found that its mitochondrial health was impaired. Notably, *sqr*^−/−^ tumor xenografts exhibited significantly slower growth compared to the *sqr*^+/+^ controls. Transcriptome and metabolome analyses indicated that SQR knockout induced a reprogramming of glycolytic flux. Our findings suggest that SQR represents a promising potential target for CRC treatment.

## 2 Results

### 2.1 Transcription of *sqr* is relatively high in colon and rectum tumors

Clinical sample analysis through the GEPIA database revealed that SQR is significantly upregulated in the vast majority of tumor tissues (Supplementary Fig. S1A-E). However, the synthesis and metabolism of H_2_S primarily occur in the human gut by gut microbiota. To check whether SQR expression is altered in CRC cells, we compared its mRNA levels in two types of CRC samples, the colon adenocarcinoma (COAD) and rectum adenocarcinoma (READ). Data from 275 COAD samples and 349 corresponding normal samples, 92 READ samples and 318 corresponding normal samples were collected and analyzed using Gene Expression Profiling Interactive Analysis (GEPIA). Our analysis revealed that the median expression levels of *sqr* in both COAD and READ tumor tissues were modestly elevated compared to those in the corresponding normal tissues (Supplementary Fig. S2A and B), suggesting that *sqr* expression is related to CRC tumor development.

### 2.2 SQR knockout leads to decreased intracellular polysulfides and dampened cell proliferation

To elucidate the function of SQR in CRC cells, we constructed HCT116 *sqr* KO cell line (*sqr*^−/−^). The exon 1 region of *sqr* was selected as the target for CRISPR/Cas9 genome editing (Supplementary Fig. S2C). Single clones were obtained and subsequently validated via PCR and DNA sequencing. PCR verification (using F1/R1 primer pair) results indicated that 106 bp of exon 1 sequence was deleted in the finally obtained *sqr*^*−/−*^ cell (Supplementary Fig. S2D). PCR with F1/R2 primer pair generated correct 481-bp DNA fragment in *sqr*^*+/+*^ but generated no fragment in *sqr*^−/−^, further confirming the deletion of 106-bp sequence (Supplementary Fig. S2D). The contents of *sqr* mRNA in *sqr*^*+/+*^ and *sqr−/−* cells were examined with RT-qPCR. No mRNA fragment containing the deleted sequence was detected, while mRNA containing undeleted sequence was detected in *sqr*^*−/−*^ cells (Supplementary Fig. S2E and F). For confirmation, we also validated the knockout effect of SQR at the protein level. Western blot results indicated that the SQR protein was undetectable in the *sqr*^*−/−*^ cell line (Supplementary Fig. S2G-J). The above results indicated that *sqr*^−/−^ cell line was successfully constructed.

**Fig. 1:**
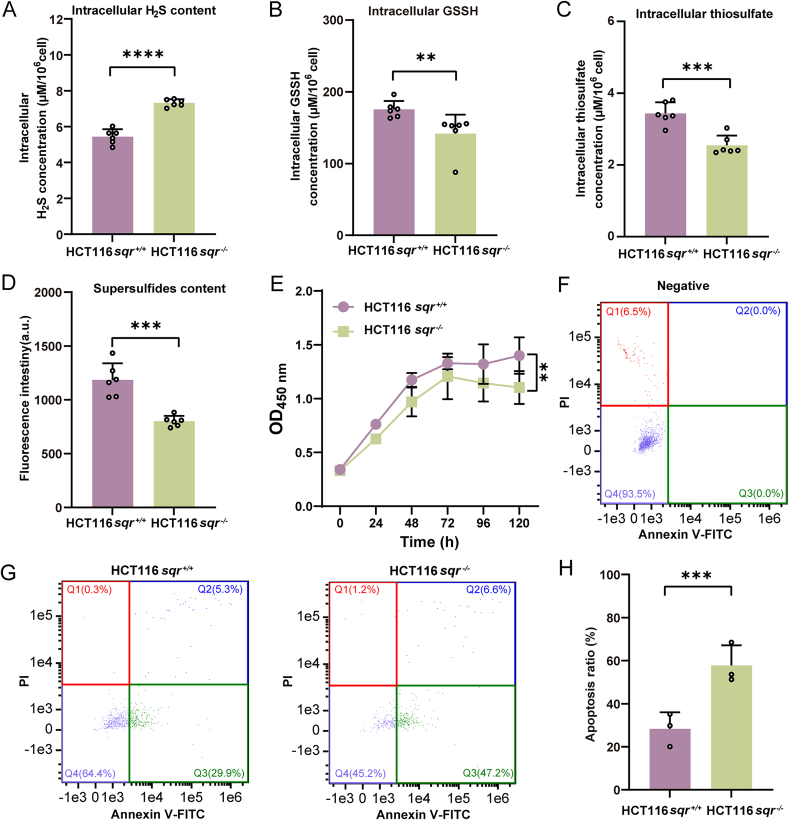
Intracellular polysulfides, cell proliferation and apoptosis analysis. (A–C) Quantification of intracellular H_2_S (A) (*n* = 6 each), GSSH (B) (*n* = 6 each), thiosulfate (C) (*n* = 6 each). (D) Quantification of total S^0^ content. S^0^ was evaluated by chemical probe SSP4 (D) (*n* = 6 each). (E) SQR knockout inhibited the proliferation of HCT116 cells (*n* = 3 each). (F–H) Cell apoptosis analysis. (F) *sqr*^+/+^ cells that were not stained with Annexin V-FITC and PI stains served as negative controls, and the distribution of single intact living cells within the group was determined using quantitative imaging. (G) Q1 (Annexin V-FITC^-^/PI^+^) represents mechanical injury cells, Q2 (Annexin V-FITC^+^/PI^+^) represents late apoptotic cells, Q3 (Annexin V-FITC^+^/PI^−^) represents early apoptotic cells and Q4 (Annexin V-FITC^-^/PI^−^) represents normal cells. (H) There were more early and late apoptotic cells in *sqr*^−/−^ than that in *sqr*^+/+^ (Q2+Q3 vs O4) (*n* = 3 each). Data were from three or six independent repeats and shown as average ± SD. The two-tailed unpaired Student's *t*-tests were performed (A), (C), (D) and (H). A two-tailed paired Student's *t*-tests was performed for (E). Mann-Whitney *U* test was performed for (B). ∗*p* < 0.05, ∗∗*p* < 0.01, ∗∗∗*p* < 0.001, ∗∗∗∗*p* < 0.0001.

SQR catalyzes the initial step in the mitochondrial sulfide oxidation pathway [16]. It oxidizes H_2_S to zero valent sulfur (S^0^), which enters the intracellular polysulfides pool [28]. To explore the impact of SQR knockout on the sulfide oxidation process, we quantified intracellular H_2_S, GSSH, and thiosulfate in both *sqr*^*+/+*^ and *sqr*^*−/−*^. Intracellular H_2_S was 5.43 ± 0.43 μM/10^6^ cells in *sqr*^*+/+*^ and 7.32 ± 0.54 μM/10^6^ cells in *sqr*^*−/−*^ (Fig. 1A). Compared with *sqr*^*+/+*^, *sqr*^*−/−*^ contained less intracellular GSSH and thiosulfate (Fig. 1B and C). Intracellular total polysulfides contents were analyzed using the fluorescence probe SSP4. Fluorescence intensity of *sqr*^*−/−*^ was significantly lower than that of *sqr*^*+/+*^ (Fig. 1D). Collectively, these findings illustrated that SQR knockout led to the decrease of H_2_S oxidation and intracellular polysulfides.

Subsequently, we cultivated HCT116 cells in McCOY's 5 A medium and observed that cell density of *sqr*^*−/−*^ was 20–30 % lower than that of *sqr*^*+/+*^ (Fig. 1E). Cell apoptosis analysis was performed using the TransDetect Annexin V-FITC/PI kit and flow cytometry. For *sqr*^*+/+*^, the percentages of late apoptotic cells and early apoptotic cells were 5.3 % and 29.9 %; whereas, these percentages were 6.6 % and 47.2 % for *sqr*^*−/−*^ (Fig. 1F–H). These data indicated that SQR knockout induced cell apoptosis.

### 2.3 SQR knockout leads to impaired mitochondrial health

**Fig. 2:**
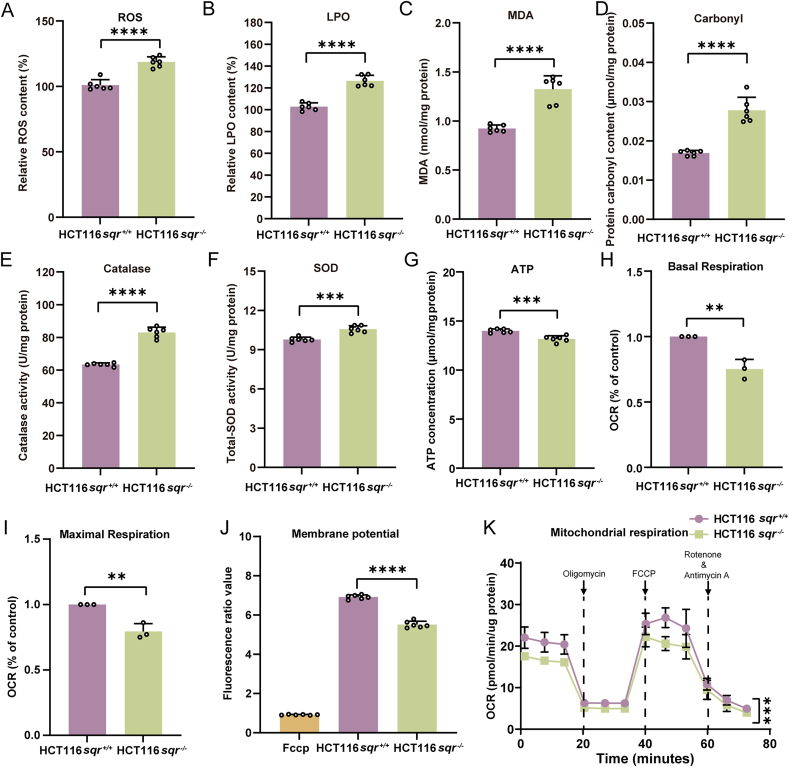
Mitochondrial physiology analysis (A-G) Intracellular ROS (A) (*n* = 6 each), LPO (B) (*n* = 6 each), MDA (C) (*n* = 6 each), protein carbonyl (D) (*n* = 6 each), Catalase activity (E) (*n* = 6 each), total SODs activity (F) (*n* = 6 each) and ATP (G) (*n* = 6 each) contents of *sqr*^+/+^ and *sqr*^−/−^ cells. (H and I) Oxygen consumption rate (OCR) analysis (*n* = 3 each). (J) Mitochondrial membrane potential of *sqr*^+/+^ and *sqr*^−/−^ cells. The *sqr*^+/+^ cells treated with 10 μM CCCP (mitochondrial uncoupling agent) was used as the positive control (*n* = 6 each). (K) Seahorse XF Cell Mito Stress test profiles of *sqr*^+/+^ and *sqr*^−/−^ cells (*n* = 3 each). Data were from three or six independent repeats and shown as average ± SD. The two-tailed unpaired Student's *t*-tests were performed (A to J). A two-tailed paired Student's *t*-tests was performed for (K). ∗*p* < 0.05, ∗∗*p* < 0.01, ∗∗∗*p* < 0.001, ∗∗∗∗*p* < 0.0001.

To evaluate the influence of SQR knockout on mitochondrial function, we conducted a comprehensive analysis of key mitochondrial physiological parameters and redox levels in HCT116 cells, including reactive oxygen species (ROS) content, lipid peroxidation (LPO) levels, LPO biomarker malondialdehyde (MDA) content, protein carbonyl levels, antioxidant enzymes catalase and total superoxide dismutase (SOD) activity, ATP production, mitochondrial membrane potential (MMP), and oxygen consumption rate (OCR). Under normal conditions, mitochondria generate ROS via the electron transport chain (ETC), a key source of cellular ROS. However, mitochondrial dysfunction can cause excessive ROS production, damaging mitochondrial proteins, lipids, and DNA, thus impairing their function. In this study, the ROS, LPO, MDA and protein carbonyl content in *sqr*^*−/−*^ was significantly higher than that in *sqr*^*+/+*^ (Fig. 2A–D). Excessive ROS stimulates an increase in the activity of antioxidant enzymes like SOD and catalase (Fig. 2E and F) to counteract intracellular adverse environment. Simultaneously, the *sqr*^*−/−*^ cells produced marginally less ATP than the *sqr*^*+/+*^ cells (Fig. 2G). MMP was also reduced in *sqr*^*−/−*^ compared with that in *sqr*^*+/+*^ (Fig. 2J). Both mitochondrial basal OCR and maximal OCR of *sqr*^*−/−*^ were lower than those of *sqr*^*+/+*^ (Fig. 2H, I and K). These results suggested that SQR knockout impaired functions of mitochondria, potentially disrupted the energy supply within the cell.

**Fig. 3:**
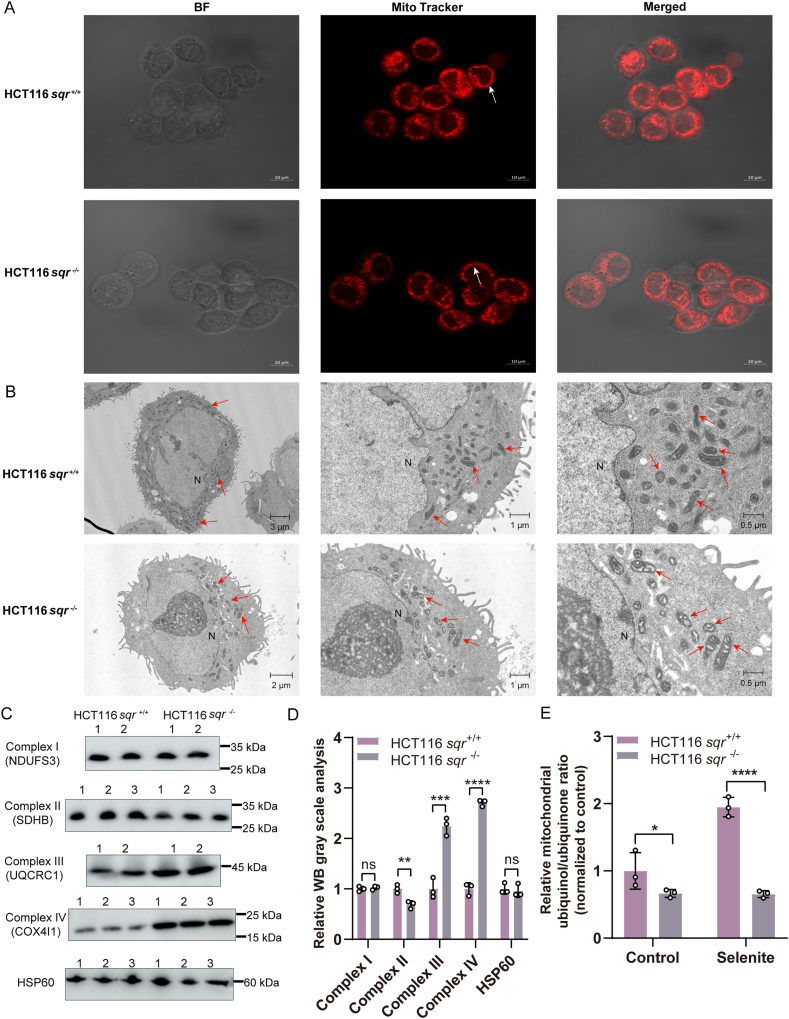
Mitochondrial morphology and functional biochemical analysis (A) Mitochondrial morphology was analyzed using Mito-Tracker Red fluorescent dye and confocal laser scanning microscope. (B) Mitochondrial morphology was observed by a transmission electron microscope (TEM). The detailed scales were marked at the bottom right corner of figures. N indicates the nucleus, and the red arrow indicates the mitochondria. (C) Mitochondrial complex subunits in cell lines were analyzed by western blotting. HSP60 protein was used as an internal control. (D) The gray values of the western blotting bands in cell lines (n = 3 each) were analyzed by Image J software. WB grayscale is the relative value of samples on the same film. (E) The ubiquinol/ubiquinone (CoQ_10_) ratio in mitochondria was determined by UHPLC-MS/MS. The *sqr*^+/+^ and *sqr*^−/−^ cells were treated with vehicle (as the control) or 10 μM selenite for 2 h. The two-tailed unpaired Student's *t*-tests were performed for (D). Mann-Whitney *U* test was performed for (E). ns: no significance, ∗*p* < 0.05, ∗∗*p* < 0.01, ∗∗∗*p* < 0.001, ∗∗∗∗*p* < 0.0001.

Mitochondrial dysfunction is commonly associated with alterations in mitochondrial morphology [29]. We utilized Mito-Tracker Red and a confocal laser scanning microscope to scrutinize the morphology of HCT116 mitochondria. Compared with mitochondria of *sqr*^*+/+*^, which displayed strong fluorescence and mostly banded shape, mitochondria of *sqr*^*−/−*^ displayed weaker fluorescence and mostly intermittent shape (Fig. 3A). To further clarify the alterations in mitochondrial morphology, we employed a transmission electron microscope (TEM) to observe HCT116 *sqr*^+/+^ and *sqr*^−/−^ cells following previously reported methods [[30], [31], [32]]. The results demonstrated that under different magnification fields of view, there were distinct differences in mitochondrial morphology between *sqr*^+/+^ cells and *sqr*^−/−^ cells. Specifically, the inter structure of *sqr*^+/+^ mitochondria is compact, while that of *sqr*^−/−^ mitochondria is loose (Fig. 3B). Western blot analyses of mitochondrial complex subunits revealed that the abundance of complex III and complex IV was significantly elevated in *sqr*^−/−^ cells, whereas the expression of complex II, which is responsible for the reduction of Coenzyme Q_10_ (ubiquinone, CoQ_10_), was markedly decreased. No significant alterations were observed in the levels of complex I or the internal reference protein HSP60, which is localized in the mitochondrial matrix (Fig. 3C and D). Furthermore, we examined the redox status of CoQ_10_ in *sqr*^+/+^ and *sqr*^−/−^ cells based on UHPLC-MS/MS [33,34], and discovered that the selenite-induced production of reduced CoQ_10_ (ubiquinol, CoQ_10_H_2_) was diminished as a result of SQR deletion (Fig. 3E). Taken together, the above observations and analyses indicated that SQR knockout impaired the heath of mitochondria.

### 2.4 SQR knockout leads to inhibited growth of the colon tumor xenograft

**Fig. 4:**
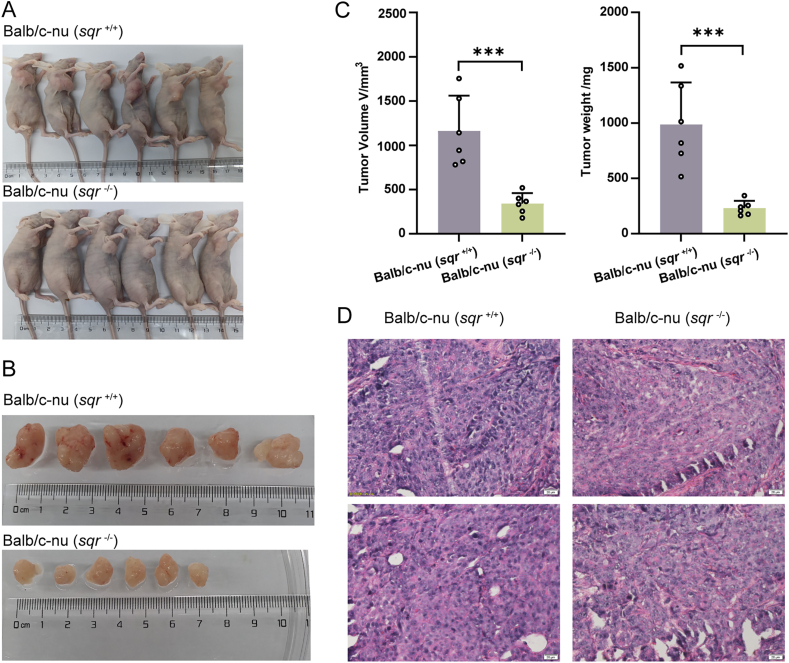
Colon tumor xenografts derived from HCT116 cells (A-C) Athymic nude female mice were inoculated subcutaneously with 3 × 10^6^*sqr*^+/+^ and *sqr*^−/−^ cells, respectively. After 3 weeks, these mice were photographed (A) (*n* = 6 each), and their tumors were dissected, photographed (B), measured and weighted (C) (*n* = 6 each). (D) H&E staining analysis of xenograft tumor tissue. These images were randomly selected from Balb/c-nu (*sqr*^+/+^) mice tissue (left) and Balb/c-nu (*sqr*^−/−^) mice tissue (right). Data were from six independent repeats and shown as average ± SD. The two-tailed unpaired Student's *t*-tests were performed (C). ∗∗∗*p* < 0.001.

To assess the impact of SQR knockout on colon tumor proliferation, we established tumor xenograft mice models with HCT116 cells. The *sqr*^+/+^ and *sqr*^−/−^ cells were individually transplanted into immune-deficient mice (Balb/c nude) by subcutaneous injection. The volumes and weights of xenograft tumors were measured after 20 days (Fig. 4A and B). Notably, we found that both indices of Balb/c-nu (*sqr*^−/−^) group were significantly lower than that of Balb/c-nu (*sqr*^+/+^) group (Fig. 4C). The average volume of *sqr*^−/−^ derived tumors only was 1/3 of the average volume of *sqr*^+/+^ derived tumors, and the average weight was less than 1/3. Histological section analysis showed that in *sqr*^+/+^ derived tumors, cells exhibited regular-shaped nuclei with uniform size distribution; whereas, in *sqr*^−/−^ derived tumors, cells displayed irregularly-shaped nuclei with pleomorphism and sparse arrangement (Fig. 4D).

### 2.5 Transcriptomic analysis of tumor xenografts indicates that SQR knockout causes a large number of gene transcriptional changes, involving multiple metabolic pathways

**Fig. 5:**
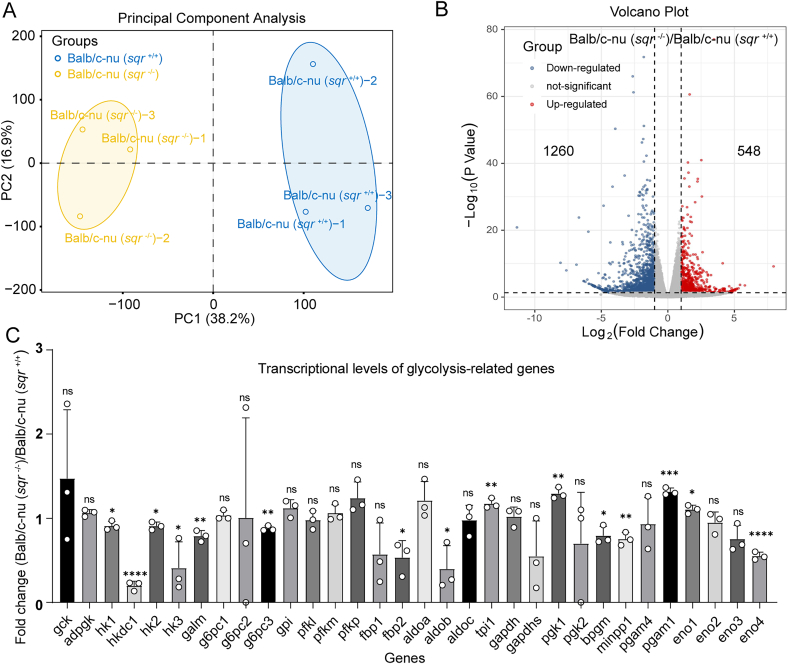
Transcriptomic analysis of HCT116 derived tumor xenografts (A) The transcriptional principal component analysis of Balb/c-nu (s*qr*^+/+^) and Balb/c-nu (*sqr*^−/−^) groups (*n* = 3 each). Dots of the same color represent individual biology replicates within the group, and the distance between the dots represents the overall expression difference of the samples. The colored ovals represent 95 % confidence intervals. (B) Differentially expressed genes were identified in Balb/c-nu (*sqr*^+/+^) and Balb/c-nu (*sqr*^−/−^) mice tissue by RNA-sequencing. (C) The transcriptional levels of genes involved in the glycolytic pathway determined by transcriptome data. The y-axis shows the fold change in expression levels of glycolytic genes in Balb/c-nu (*sqr*^+/+^) mice tissue over the levels in Balb/c-nu (*sqr*^−/−^) mice tissue (*n* = 3 each). Detailed information on gene abbreviations is shown in Table S1. The two-tailed unpaired Student's *t*-tests were performed for (C). ns: no significance, ∗*p* < 0.05, ∗∗*p* < 0.01, ∗∗∗*p* < 0.001, ∗∗∗∗*p* < 0.0001.

Since polysulfides are involved in a myriad of physiological processes and SQR knockout significantly decreased the content of intracellular polysulfides, we suspected that SQR knockout may result in a vast change of transcription profile. Therefore, transcriptome sequencing (RNA-seq) was performed on *sqr*^+/+^ and *sqr*^−/−^ derived tumors. According to the transcriptional principal component analysis (PCA) of *sqr*^+/+^ and *sqr*^−/−^ groups, the intergroup samples were dispersed while the intra-group samples were clustered together, suggesting that there were significant differences between the two groups (Fig. 5A). We totally identified 1800 genes with apparent transcriptional changes (fold change>2, p < 0.05) in *sqr*^−/−^ xenografts in comparison with *sqr*^+/+^ xenografts, of which 1260 genes were down-regulated and 548 genes were up-regulated (Fig. 5B). Since cancer cells commonly exhibit the Warburg effect [35], we initially focused on the changes of glycolytic related genes but found that expressions of most glycolytic genes were not affected (transcriptional changes<1.5-fold), with glucokinase (GCK) displaying the most prominent change at 1.47-fold. Additionally, phosphoglycerate mutase 1 (PGAM1) and phosphoglycerate kinase 1 (PGK1) displayed increased fold changes of 1.32 and 1.29 respectively (Fig. 5C and Supplementary Tab S1). Although the transcription levels of phosphofructokinase (PFKP) and aldolase (ALDOA) were detected to vary by 1.24 and 1.20 times, statistical analysis revealed no significance. Overall, transcriptome analysis revealed that SQR knockout had limited impact on the expression of glycolytic genes.

To investigate the effect of SQR knockout on other cell processes, we performed Gene Ontology (GO) and Kyoto Encyclopedia of Genes and Genomes (KEGG) enrichment analysis. The GOMF (Gene Ontology Molecular Function) related processes were significantly enriched along with other processes, and extrinsic component of mitochondrial outer membrane occupied the highest rich factor, no pathway directly linked to sulfur or glucose metabolism was identified (Supplementary Fig. S3). GO analysis indicated that transcriptional regulation related cell processes were enriched, suggesting that SQR knockout led to transcription profile alteration in tumor cells (Supplementary Fig. S3). KEGG analysis showed that the top 4 changed processes were maturity onset diabetes of the young, arginine biosynthesis, circadian rhythm, and staphylococcus infection (Supplementary Fig. S4). Gene Set Enrichment Analysis (GSEA) of the WiKi pathway dataset indicated that the top 4 (Supplementary Fig. S5A-D) pathways were retinoblastoma genes in cancer, proteasome degradation, electron transport chain oxidative phosphorylation system in mitochondria, and the TCA cycle. In addition, No.11 (Supplementary Fig. S5E) and No.15 (Supplementary Fig. S5F) pathways with significant enrichment were aerobic glycolysis and sulfation biotransformation reaction, respectively.

### 2.6 Metabolomics analysis of tumor xenografts shows that SQR knockout results in changes in the content of multiple metabolites, especially glycolytic metabolites

**Fig. 6:**
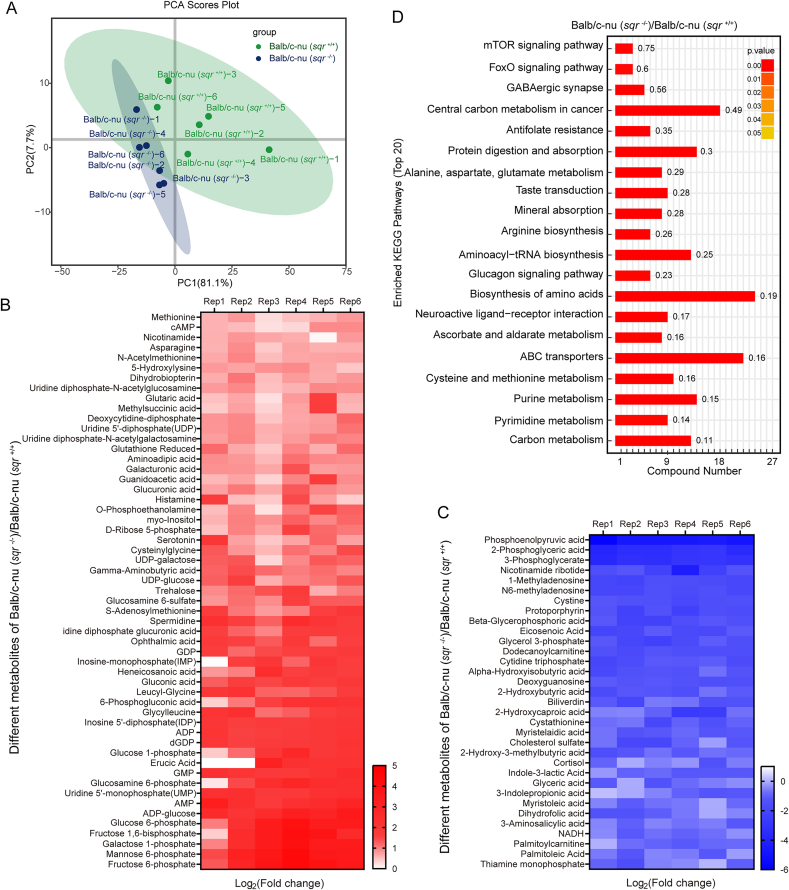
Metabolomics analysis of tumor xenografts (A) Principal component analysis of Balb/c-nu (*sqr*^+/+^) and Balb/c-nu (*sqr*^−/−^) samples (*n* = 6 each). The colored ovals represent 95 % confidence intervals. (B and C) Content increased (B) (*n* = 6 each) and decreased (C) (*n* = 6 each) metabolites with fold Change>1.5. (D) Top 20 enriched KEGG pathways reflected by the content changed metabolites.

Targeted metabolomics assay was performed on *sqr*^+/+^ and *sqr*^−/−^ derived tumors. PCA scores plot revealed that samples of the same tumor group exhibited similar patterns (Fig. 6A), demonstrating high representativeness and reproducibility of the samples. Totally 364 metabolites were identified, among which 55 metabolites were increased and 33 were decreased (fold change>1.5, p < 0.05) (Fig. 6B and C). The changed metabolites encompassed organic phosphoric acids and derivatives, organooxygen compounds, pyridine nucleotides, purine nucleosides, carboxylic acids and derivatives and so on, which cover a wide range of known metabolic pathways (Supplementary Fig. S6 and Tab. S2). KEGG analysis indicated that central carbon metabolism, amino acid biosynthesis pathways, and ABC transport system displayed substantial alterations (Fig. 6D). Cysteine and methionine metabolism, which is closely linked with polysulfides metabolism, was also changed.

**Fig. 7:**
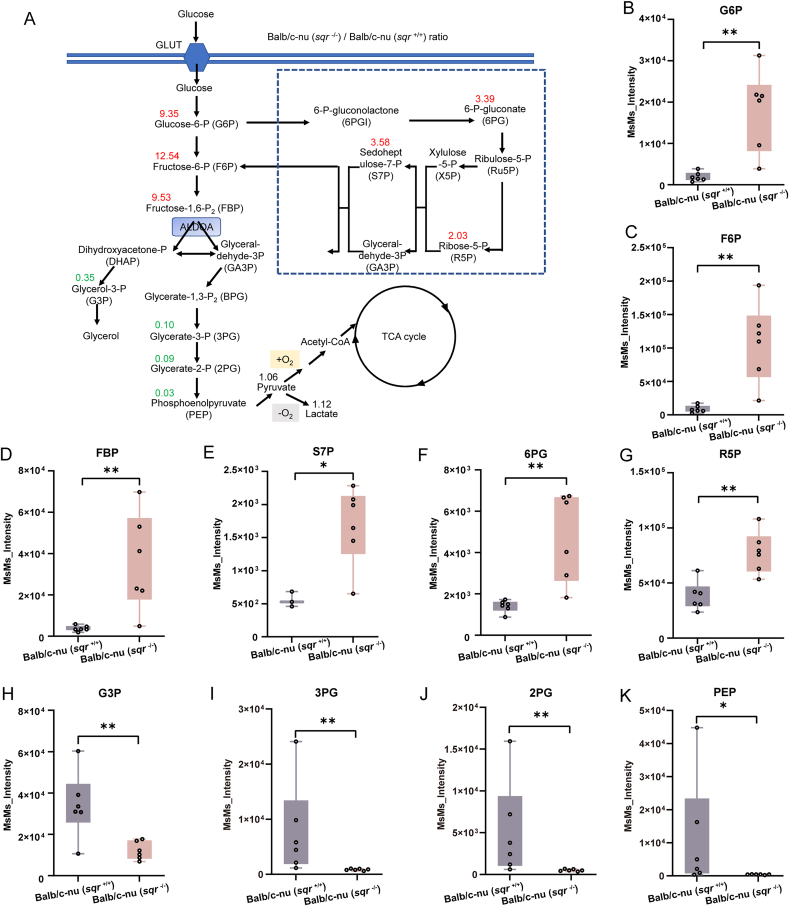
Content change of metabolites in glycolysis pathway (A) Content changed metabolites in glycolytic and pentose phosphate pathways owing to SQR knockout. The numbers represent content ratios of Balb/c-nu (*sqr*^−/−^)/Balb/c-nu (*sqr*^+/+^). (B–K) Detailed data of content changed metabolites. (B) G6P, glucose-6-phosphate (n = 6 each); (C) F6P, fructose-6-phosphate (n = 6 each); (D) FBP, fructose 1,6-bisphosphate (n = 6 each); (E) S7P, sedoheptulose-7-phosphate (n = 3, 6); (F) 6 PG, 6-phosphogluconic acid (n = 6 each); (G) R5P, ribose 5-phosphate (n = 6 each); (H) G3P, glycerol-3-phosphate (n = 6 each); (I) 3 PG, 3-phosphoglyceric acid (n = 6 each); (J) 2 PG, 2-phosphoglyceric acid (n = 6 each); (K) PEP, phosphoenolpyruvate (n = 6 each). Data were from six independent repeats and shown as average ± SD. The two-tailed unpaired Student's *t*-tests were performed for (B to H). Mann–Whitney *U* tests were performed for (I to K). ∗*p* < 0.05, ∗∗*p* < 0.01, ∗∗∗*p* < 0.001.

Upon ranking the metabolites by their fold changes, we found that most of those with the largest changes were related to glycolysis (Fig. 7A). Specifically, contents of glucose-6-phosphate (G6P), fructose-6-phosphate (F6P), and fructose 1,6-bisphosphate (FBP) increased 9.35, 12.54 and 9.53 folds, respectively in *sqr*^−/−^ derived tumor (Fig. 7B–D). Contents of three pentose phosphate pathway metabolites, sedoheptulose-7-phosphate (S7P), 6-phosphogluconic acid (6 PG), and ribose 5-phosphate (R5P), increased 3.58, 3.39, and 2.03 folds, respectively (Fig. 7E–G). Whereas, contents of 3-phosphoglyceric acid (3 PG), 2-phosphoglyceric acid (2 PG), phosphoenolpyruvate (PEP), and glycerol-3-phosphate (G3P) reduced 0.10, 0.09, 0.03, and 0.35 folds, respectively (Fig. 7H–K). Therefore, with FBP as the boundary, upstream metabolites were accumulated while downstream metabolites were consumed. These results suggested that the glycolic flux was clogged in FBP breakdown step in *sqr*^−/−^ derived tumors.

It is noteworthy that further downstream flux was not impacted by SQR knockout, evidenced by similar pyruvate and lactate contents between *sqr*^−/−^ and *sqr*^+/+^ derived tumors. TCA cycle metabolites also showed no significant change except for α-ketoglutarate and malate, whose concentration increased 1.48 and 1.29 folds (Supplementary Fig. S7).

### 2.7 SQR knockout decreases content of ALDOA enzyme

**Fig. 8:**
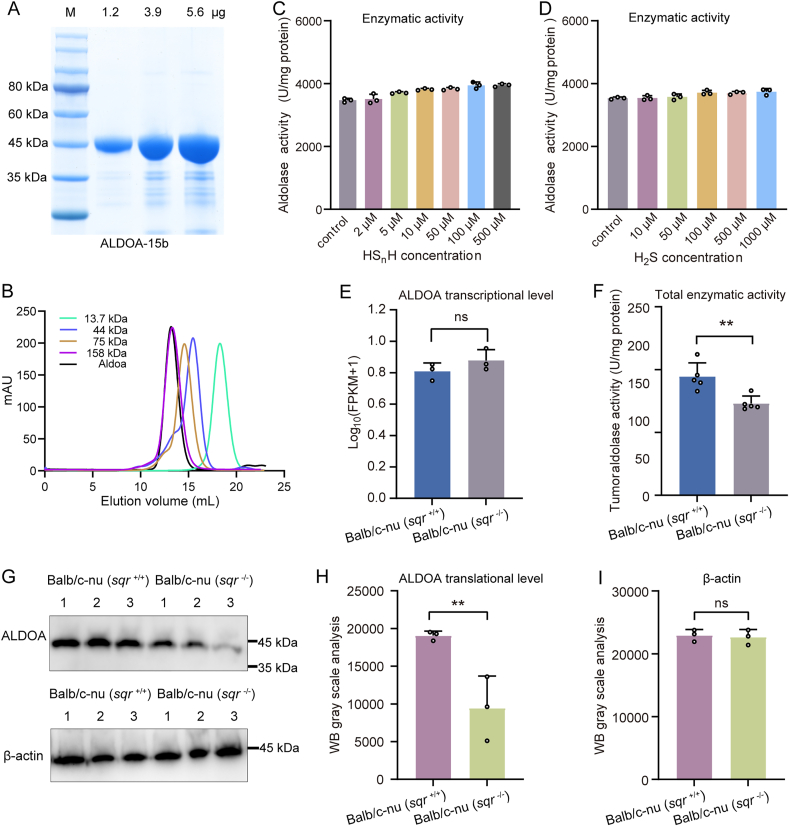
ALDOA purification, activity determination, and expression analysis (A) SDS-PAGE analysis of purified ALDOA. (B) Size-exclusion chromotography analysis of purified ALDOA. (C and D) Enzyme activity analysis was performed by treating ALDOA with HS_n_H (C) (n = 3 each) or H_2_S (D) (n = 3 each). (E) Transcription level of ALDOA in HCT116 derived tumors (n = 3 each). (F) Enzyme activity of ALDOA determined with protein extract of tumor xenografts (n = 5 each). (G) ALDOA protein level in Balb/c-nu (*sqr*^+/+^) and Balb/c-nu (*sqr*^−/−^) tumors were analyzed by western blotting. β-actin protein was used as an internal control. (H and I) The gray values of the western blotting bands were analyzed by Image J software (n = 3 each). Data were from multiple independent repeats and shown as average ± SD. The two-tailed unpaired Student's *t*-tests were performed for (C), (D), (E), (F), (H) and (I). ns: no significance, ∗*p* < 0.05, ∗∗*p* < 0.01.

The enzyme ALDOA facilitates the breakdown of FBP into dihydroxyacetone phosphate (DHAP) and glyceraldehyde 3-phosphate (GA3P). Previous studies indicated that activity of glycolysis enzyme can be affected by polysulfides via the posttranslational modification—protein persulfidation (protein-SSH) [36,37]. We suspected a similar effect on ALDOA. To test this hypothesis, we expressed the HCT116 ALDOA encoding gene in *E. coli* BL21 (with *N*-terminal His tag) and purified this enzyme with a Nickle column. SDS-PAGE analysis showed that the molecular weight of ALDOA monomer was around 45 kDa (the theoretical molecular weight is 41 kDa) and size-exclusion chromotography analysis revealed that the peak of freshly purified ALDOA coincided with that of 158-kDa standard (Fig. 8A and B), indicating that purified ALDOA existed in tetramer form, which was consistent with previous reports [38].

We then used HS_n_H to treat purified ALDOA, but found that HS_n_H (5–500 μM) had no obvious influence on ALDOA. ALDOA activity only increased 1.13-fold at 500 μM HS_n_H condition (Fig. 8C). We also used H_2_S to treat ALDOA and observed that its activity was not obviously changed either (Fig. 8D). Therefore, we concluded that ALDOA activity was not directly influenced by polysulfides or H_2_S.

Transcriptome analysis indicated that the transcription level of ALDOA had no obvious change in *sqr*^−/−^ tumor (1.21-fold) (Fig. 8E). However, when we measured ALDOA activity in crude enzyme extracts obtained from xenograft tumors, we observed lower activity in the *sqr*^−/−^ derived tumor than that in the *sqr*^+/+^ derived tumor (Fig. 8F). Western blot analysis further revealed that the ALDOA protein content was significantly reduced in *sqr*^−/−^ derived tumors (Fig. 8G–I). Collectively, these results indicated that SQR knockout resulted in decreased content of the ALDOA enzyme.

A previous study indicated that ALDOA can be S-persulfidated and this posttranslational modification might affect the stability of the protein [12]. We identified the presence of S-persulfidation on Cys^72^ and Cys^239^ residues of HS_n_H treated ALDOA. These modifications were not detected in the DTT or H_2_S treated groups (Supplementary Fig S8A). The original peak chart corresponding to the mass spectrometry data can be found in the Supplementary Materials (Supplementary Fig. S9A-C and Fig. S10A-C). We analyzed the three-dimensional structure of ALDOA monomer (PDB: 4ald) and observed that Cys^72^ and Cys^239^ were located on the protein surface, which give them the chance to react with polysulfides (Supplementary Fig. S8B-D).

### 2.8 The SQR inhibitor effectively suppressed growth of colon tumor xenografts

**Fig. 9:**
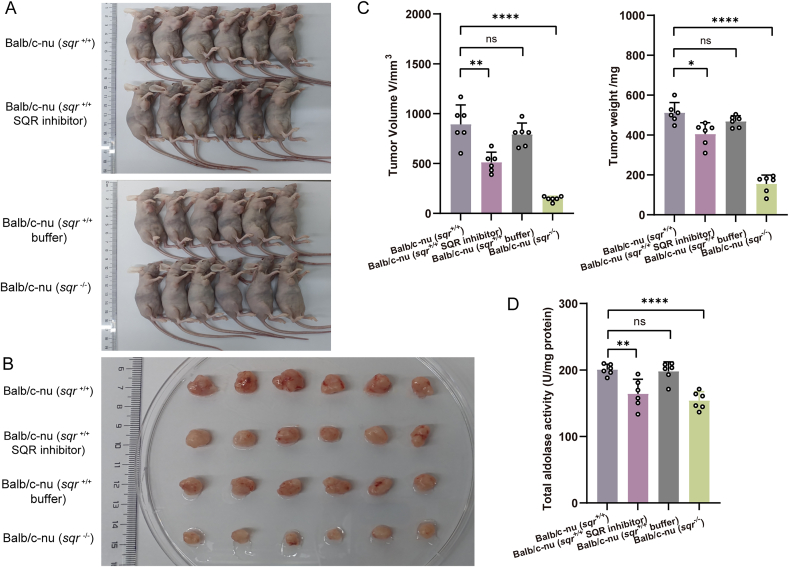
The SQR inhibitor FC9402 affected growth of colon tumor xenografts (A-C) Balb/c nude mice were randomly divided into four groups. Three groups were injected with *sqr*^+/+^ cells, while one group was injected with *sqr*^−/−^ cells. Among the groups receiving *sqr*^+/+^ cells, two groups were intraperitoneally administered either the FC9402 or solution buffer (as a control) every three days. After 20 days, all mice were photographed (A) (n = 6 for each group), and their tumors were excised, photographed (B), measured, and weighed (C) (n = 6 for each group). (D) Enzyme activity of ALDOA was determined using protein extracts from tumor xenografts (n = 6 for each group). Data were from six independent repeats and shown as average ± SD. The two-tailed unpaired Student's *t*-tests were performed (C and D). ns: no significance, ∗*p* < 0.05, ∗∗*p* < 0.01, ∗∗∗*p* < 0.001, ∗∗∗∗*p* < 0.0001.

To further substantiate the association between SQR expression and the growth of colon tumors, we used the SQR inhibitor FC9402 to treat the tumor xenograft mouse models [39]. Immunodeficient Balb/c nude mice were divided into four groups: three groups injected with *sqr*^+/+^ cells and one group injected with *sqr*^*−/−*^ cells. Following xenotransplantation, one group of mice harboring *sqr*^*+/+*^ cells were not treated (as the first control); one group of mice harboring *sqr*^*+/+*^ cells were intraperitoneally administered with the injection solvent buffer (as the second control group); one group of mice harboring *sqr*^*+/+*^ cells were treated with FC9402 (5 μg per 10 g body weight, per 3 days). After 20 days, the xenograft tumor volume and weight were assessed. Results indicated that both the tumor volume and weight of FC9402 treated group were significantly reduced in comparison to that of two control groups, but were still higher than that of *sqr*^*−/−*^ group (Fig. 9A–C). These results confirmed that SQR activity affected the colon tumor growth. Subsequently, crude enzyme extracts were prepared from the aforementioned tissues and ALDOA activity was measured. ALDOA activity in FC9402 treated group was reduced in comparison to that of two control groups (Fig. 9D). Collectively, these data suggest that SQR may serve as a promising drug target for the CRC treatment.

## 3 Discussion

As a specific polysulfides producing enzyme located in mitochondria, SQR is expected to play important roles in CRC development [40]. In this study, we constructed the SQR knockout cell line of HCT116 and found that its cell proliferation rate was decreased. Mitochondria exhibited impaired physiological functions and abnormal morphology. Furthermore, we observed that SQR knockout significantly restrained the development of colon tumor xenograft, evidenced by the volume and weight of *sqr*^−/−^ derived tumors both decreased about 2/3. Histological section analysis indicated that in *sqr*^−/−^ derived tumors, cells displayed irregularly-shaped nuclei and sparse arrangement. Although transcriptions of glycolysis related genes were not obviously changed, metabolome analysis indicated that the glycolysis pathway was clogged in the step of FBP degradation, which is catalyzed by the ALDOA enzyme. ALDOA content was decreased and its activity was reduced in *sqr*^−/−^. Overall, we concluded that SQR knockout led to mitochondrial dysfunction and metabolic reprogramming, thereby inhibited the growth of HCT116 tumor xenograft.

Previous studies indicated that SQR can alleviate brain damage caused by hypoxia, with its expression level inversely correlating to the brain's sensitivity to hypoxia, positioning SQR as a potential therapeutic target for ischemic brain injury [41]. SQR is also involved in ferroptosis and acute kidney injury (AKI). It is highly expressed in renal cortex. The ubiquitin-mediated degradation of SQR could induce mitochondrial dysfunction, thus accelerating ferroptosis and occurrence of AKI [42]. Our study has unveiled critical roles of SQR in CRC tumor development. The diverse functions of SQR may contribute to its involvement in polysulfides production, given numerous reports illustrating the potency of polysulfides as intracellular compounds involved in various cellular processes [[43], [44], [45]]. However, the underlying mechanisms of polysulfides action remain elusive, and research on intracellular polysulfides is still in its infant stage.

ALDOA catalyzes the cleavage of FBP to GA3P and DHAP. There are three aldolase isozymes in humans, named aldolase A, B, and C, which are differentially expressed in tissues and have been linked to various diseases and cancers [46]. Notably, elevated levels of ALDOA have been reported in tumor cells and cancer patient samples, including those from oral squamous cell carcinoma, osteosarcoma, hepatocellular cell carcinoma, and lung cancer [47]. It is believed that ALDOA plays additional roles beyond FBP cleavage. For instance, ALDOA has been shown to promote liver cancer growth and metastasis by accelerating mRNA translation and subsequently enhancing overall protein biosynthesis. Inhibiting ALDOA effectively slows the growth of orthotopic xenografts [38]. Herein, we observed that ALDOA content was reduced in HCT116 *sqr*^−/−^ derived tumor xenografts, which should be one of the reasons why growth of tumor xenografts was significantly decreased. However, SQR knockout didn't affect the transcription level of ALDOA encoding gene, implying that the ALDOA content reduction may be due to decreased translation efficiency or shortened protein half-life.

While mitochondrial respiration is more efficient in energy production, rapidly growing cancer cells favor aerobic glycolysis, known as the Warburg effect [48,49]. This phenomenon is generally thought to be due to the faster production of ATP by per unit mass glycolytic proteins. But recent research suggests that the mitochondrial respiration proteome is more efficient, and that cells perform aerobic glycolysis to meet acute energy needs and prepare for energy-limiting harsh environments such as hypoxia [50]. There also is an emerging idea that mitochondrial metabolism is not suppressed in tumors, but its activity reaches supersaturated levels [51]. Therefore, mitochondria are important mediators of tumorigenesis, and understanding the mechanism of mitochondrial function during tumorigenesis will be crucial for future cancer treatment [[52], [53], [54], [55]]. It should be noted that mitochondria regulate their size, quantity, morphology and distribution in cells through division and fusion to maintain mitochondrial health and function [56]. Whether SQR knockout leads to abnormal mitochondrial division and fusion was not explored in this study.

Jorns and colleagues identified an effective inhibitor of human SQR (SQR-targeted inhibitor 1, STI1) through high-throughput screening of the small molecule compound library. STI1 inhibits SQR activity by binding with its coenzyme Q-binding pockets. This compound shows low cytotoxicity and effective myocardial therapy in mice [57,58]. Our findings suggest that STI1 can also be applied to treat CRC. Specific inhibition of key enzymes in glycolysis is a new strategy for tumor therapy [[59], [60], [61]]. Since SQR is associated with the ALDOA content, development of targeted ALDOA drugs in combination with SQR inhibitors may enhance therapeutic efficacy for CRC.

In conclusion, we found SQR knockout led to mitochondrial dysfunction and metabolic reprogramming, affect ALDOA content and enzymatic activity. SQR is an important participant in the mitochondrial regulation of glycolytic metabolism, providing data for subsequent targeted drug therapy.

## 4 Materials and methods

### 4.1 Cells lines

The HCT116 cell line, sourced from ATCC, was utilized in this study. Both the HCT116 *sqr*^+/+^ (wild-type) and HCT116 *sqr*^−/−^ (knockout) cell lines were maintained in McCoy's 5 A medium supplemented with 10 % fetal bovine serum. These cultures were incubated at 37 °C in a controlled environment with a 5 % CO_2_ atmosphere.

### 4.2 Animal studies

The animal studies were conducted with the consent of the Institutional Animal Care and Use Committee (IACUC) at University of Health and Rehabilitation Sciences, with the assigned approval number KFDX20243001. Female Balb/c-nu mice, aged 4 weeks and acquired from Charles River, China, were inoculated subcutaneously with 0.2 ml (3 × 10^6^ cells) HCT116 *sqr*^+/+^ or HCT116 *sqr*^−/−^ cells. After a 3-week period for tumor growth, colon tumor xenograft tissues were harvested under the auspices of the relevant project authorizations. Tumor volumes were calculated with the formula: volume (mm^3^) = (width)^2^ × length × 0.52. The weight of the tumor was measured by an analytical balance. In addition, tumor tissue samples were sent to the company for transcriptomic and targeted metabolomics analysis. FC9402 (SQR inhibitor) was purchased from MCE (HY-141552), Stock solutions of FC9402 (10 mg/ml) were prepared in DMSO and stored at −80 °C for subsequent use. The injection vehicle consisted of 0.9 % sodium chloride solution containing 40 % PEG300, 5 % Tween-80, and 1 % DMSO, with (FC9402-containing solution) or without FC9402 (FC9402-free solution, as a control), respectively. To establish the injection inhibitor model, mice were administered either FC9402-containing solution (5 μg/10 g body weight) or FC9402-free solution via intraperitoneal injection every three days over a period of three weeks, with the first injection occurring on the third day post-cell injection.

### 4.3 Reagents and antibodies

SulfoBiotics-SSP4 (cat#1810731-98-6) probe was purchased from Dojindo (Kumamoto, Japan). Sodium hydrosulfide (NaHS, cat#13590), monobromobimane (cat#B4380), thiosulfate (cat#909173), reduced glutathione (GSH, cat#G6529), carbonyl cyanide 3-chlorophenylhydrazone (CCCP, cat#C2759), hematoxylin (cat#1.04302) and eosin (cat#586X) were purchased from Sigma-Aldrich (Saint Louis, MO, USA). Assay kit of ATP, MDA, LPO, Catalase and total SOD were purchased from Beyotime Biotechnology Co., Ltd (Shanghai, China). Lipofectamine 3000 (cat#L3000150),Pierce™ BCA Protein Assay Kits and puromycin (cat#A1113802) were purchased from Thermo Fisher Scientific (Waltham, MA, USA). TURBO™ DNA-free Kit, M-MLV Reverse Transcriptase, Random Primers and MitoTracker™ Red CMXRos were purchased from Invitrogen (Carlsbad, CA, USA). TB Green® Premix Ex Taq™ II (Tli RNaseH Plus) was purchased from TaKaRa (Tokyo, Japan). XF Cell Mito Stress Test Assay Kit was purchased from Agilent Technologies (Santa Clara, CA, USA). Annexin V-FITC/PI Cell Apoptosis Detection Kit was purchased from TransGen Biotechnology (Beijing, China). Mitochondrial membrane potential assay kit with JC-1, DCFH-DA probe and Rosup (cat#S0033S) were purchased from Beyotime Biotechnology Co., Ltd (Shanghai, China). Protein Carbonyl Content Assay Kit and Fructose-bisphosphate aldolase (FBA) Activity Assay Kit were purchased from Solarbio Science & Technology Co., Ltd (Beijing, China). Superdex 200 10/300 GL Increase column and PD-10 desalting column were purchased from GE Healthcare (Little Chalfont, Buckinghamshire, UK). Ni-NTA agarose was purchased from Cytiva (Uppsala, Sweden). Anti-ALDOA Antibody (cat#A05022-3), Anti-SQOR Antibody (cat#A31817), Anti-NDUFS3 Antibody (cat#BM5340), Anti-SDHB Antibody (cat#BM5128), Anti-UQCRC1 Antibody (cat#A06974-1), Anti-COX4I1 Antibody (cat#A05442-2) and Anti-HSP60/HSPD1 Antibody (cat#BA1511) were purchased from Boster Biological Technology (Wuhan, China.). Anti-β-actin antibody (cat#A5441) was purchased from Sigma-Aldrich (Saint Louis, MO, USA). Goat Anti-Mouse IgG H&L (HRP) (cat#ab205719) and Goat Anti-Rabbit IgG H&L (HRP) (cat#ab6721) were purchased from Abcam (Cambridge, MA, USA).

### 4.4 Generation of sqr^−/−^ cells

*Sqr*^−/−^ cell were obtained using CRISPR/Cas9 genome editing. Single guide RNAs (sgRNAs) were designed using the online CRISPR design tool (https://en.rc-crispr.com/). A ranked list of sgRNAs was generated based on specificity and efficiency scores. The pair of oligos for two targeting sites were annealed and ligated to the YKO-RP006 vector (Ubigene Biosciences, Guangzhou, China). The YKO-RP006-hRABL6 [gRNA] plasmids containing each target sgRNA sequences were transfected into HCT116 cells with Lipofectamine 3000 (Thermo Fisher Scientific). For cell screening, puromycin was added after 24–48 h of transfection. After antibiotic selection, a certain number of cells were diluted using the limited dilution method and inoculated into a 96-well plate. Single clones were selected after 2–4 weeks, the cells were verified using three primers in the same PCR reaction. Forward primer *sqr* KO–F1 and two different reverse primers *sqr* KO-R1 and *sqr* KO-R2 (Supplementary Tab. S3). The wild type *sqr* allele was 936 bp, and mutant allele was 829 bp.

### 4.5 Determination of intracellular sulfane sulfur by SSP4 fluorescent probe

The sulfane sulfur content of HCT116 *sqr*^+/+^ and HCT116 *sqr*^−/−^ cells were detected using the SSP4 fluorescence probe. In short, equal number of cells were collected, washed with and re-suspended in PBS buffer (10 mM, pH 7.4). SSP4 (10 μM) and CTAB (500 μM) were added to each sample, and then the mixture was incubated at 37 °C for 15 min in the dark with gently shaking. After the incubation, cells were washed with PBS buffer (10 mM, pH 7.4) to remove excess unreacted reagents. Fluorescence intensity was measured using the SynergyH1 microplate reader. The excitation and emission wavelength were set at 482 nm and 515 nm, respectively. The final date was converted to fluorescence intensity per 10^6^ cells.

### 4.6 Cell proliferation assay

Cell proliferation assay was determined using Cell Counting kit-8 (Beyotime Biotechnology, C0037). Briefly, a certain number of cells were resuspended in culture medium, and evenly seed them into a 96-well plate with 100 μl per well, containing 2 × 10^3^ cells per well. The wells in the outermost circle of the 96-well plate are prone to evaporation, so they should be discarded and not used. After the cells have adhered to the plate, the first time test is conducted. First, remove the culture medium from the wells to be tested, then add culture medium containing 10 % (v/v) CCK8 without serum, and incubate in a 37 °C incubator for 2 h. After incubation, measure the OD_450_ of the test wells. Repeat this process, testing continuously for 5 days, and plot the curve.

### 4.7 Quantification of intracellular sulfide, GSSH, and thiosulfate by high-performance liquid chromatography (HPLC)

A previously reported fluorescence-based HPLC method was used for H_2_S, GSSH, and thiosulfate detection [62]. Briefly, cells were collected and re-suspended in reaction buffer (50 mM Tris-HCl, pH 9.5, 1 % Triton X-100, 50 μM DTPA). Depending on the experimental requirements, DTT or sulfite was added as previously described [63], followed by incubation at 95 °C for 10 min. After the incubation, the mixture was centrifuged and 50 μl supernatant was pipetted out, which was reacted with 5 μl monobromobimane (mBBr, 25 mM). The mBBr derived H_2_S, GSSH and thiosulfate were analyzed with HPLC. Based on the HPLC peak areas of H_2_S, GSSH, and thiosulfate at various concentrations, standard curves were constructed, and the concentrations of H_2_S, GSSH, and thiosulfate in the samples were subsequently determined.

### 4.8 Cellular ROS analysis

Equal number of cells were harvested and washed with PBS buffer (10 mM, pH 7.4), and then resuspended in 1 ml PBS buffer containing 10 μM dichlorofluorescein diacetate (DCFH-DA) probe. The samples were incubated at 37 °C for 20 min in the dark. After that, the samples were washed twice with PBS buffer to remove excess DCFH-DA and suspended in 1 ml PBS buffer. The fluorescence intensity of 200 μl supernatant was measured using the SynergyH1 microplate reader. The excitation and emission wavelength were set at 488 nm and 525 nm respectively. Rosup (Beyotime Biotechnology, S0033S) is a reagent mixture that functions as a potent inducer of ROS. In this experiment, cells treated with Rosup were utilized as the positive control.

### 4.9 Cellular MDA and LPO analysis

MDA and LPO levels were determined using corresponding assay kits (Beyotime Biotechnology), following the manufacturer's protocol. Cells were harvested, washed, and lysed; MDA was assayed by reacting lysates with 0.1 % thiobarbituric acid (TBA) at 100 °C for 20 min, then measuring absorbance at 532 nm. MDA content was quantified against a standard curve. For LPO, cells were stained with BODIPY 581/591C11, incubated at 37 °C for 30 min, and fluorescence of reduced and oxidized products was measured at 535/610 nm and 400/510 nm, respectively, with results expressed per milligram of protein.

### 4.10 Cellular protein carbonyl analysis

Cells were harvested in PBS, sonicated, and centrifuged. 1/10 volume of streptomycin sulfate solution (final concentration 0.01 g/mL) was added to the supernatant to eliminate nucleic acid interference, followed by centrifugation and reaction with 2 mM 2,4-dinitrophenylhydrazine (DNPH) (dissolved in 2 N HCl) at 37 °C for 60 min in the dark to form DNP derivatives. A negative control was also set with 2 N HCl. Precipitated DNP derivatives were washed with ethanol and ethyl acetate (1:1), dissolved in 6 M guanidine hydrochloride, and their absorbance at 370 nm was measured. Based on the absorbance value and the standard curve, the content of protein carbonyls per milligram of protein sample was calculated.

### 4.11 Cellular catalase and total SODs activity assay

Catalase and total superoxide dismutases (SODs) activities were determined using the corresponding assay kits (Beyotime Biotechnology). After cell collection, the measurement procedures were carried out according to the corresponding instructions, and the enzyme activities of catalase and total SODs per milligram of protein in each sample were calculated separately.

### 4.12 Cellular ATP analysis

Cellular ATP was quantified using the ATP assay kit (Beyotime Biotechnology S0026) following instructions of the manufacturer. Briefly, equal number of cells were harvested and washed with PBS buffer, and suspended in 500 μl ATP assay lysate. The cell solution was incubated at 4 °C for 5 min and then was centrifuged at 12,000 *g* for 5 min. After the centrifugation, 20 μl supernatant was pipetted out and mixed with 100 μl ATP working solution. Luminescence of the solution was immediately detected. The protein concentrations of samples were detected by Pierce™ BCA Protein Assay Kits (Thermo Fisher Scientific).

### 4.13 Mitochondrial membrane potential detection

The fluorescent probe JC-1 assay kit (Beyotime Biotechnology, C2006) was used. Equal number of cells were harvested and washed twice with PBS buffer (10 mM, pH 7.4), and then 1 ml JC-1 staining working solution was added into the dish. JC-1 probe was added following the manufacturer's instruction. After being incubated in a 5 % CO_2_, 37 °C incubator for 20 min, the cells were washed and suspended with JC-1 staining working solution. At high mitochondrial membrane potential, JC-1 accumulates in the matrix of mitochondria and forms J-aggregates, which can produce red fluorescence, the excitation and emission wavelength of J-aggregates were set at 525 and 590 nm, at low mitochondrial membrane potential, JC-1 could not accumulate in the matrix of mitochondria, and JC-1 was a monomer and could produce green fluorescence, the excitation and emission wavelength of monomer were set at 490 and 530 nm. The relative ratio of red and green fluorescence is used to measure the mitochondrial membrane potential. Mitochondrial electron transport chain inhibitor CCCP (10 μM) was used to treat cells to make a control groups.

### 4.14 Cellular OCR assay

OCR assay was performed with XF Cell Mito Stress Test Assay kit (Agilent Technologies, 103,015–100). Firstly, HCT116 *sqr*^+/+^ and HCT116 *sqr*^−/−^ cells were seeded on 96-well seahorse plates at a density of 4 × 10^4^ cells/80 μl per well. Four background correction wells contained only supplemented medium. The cell culture plate was left on the super-clean worktable for 20 min to allow the cells to settle naturally, and then plates were cultured for overnight in a 5 % CO_2_, 37 °C incubator. The probe plates were hydrated with XF Calibrant and placed in a CO_2_-free 37 °C incubator for overnight. Before the assay, cells were washed and incubated with 180 μl of glucose (10 mM), pyruvate (1 mM), and glutamine (2 mM) supplemented XF assay buffer and then plates were placed in a CO_2_-free incubator for 50 min. At the same time, oligomycin (1.5 μM), rotenone & antimycin A (1.0 μM), and FCCP (1.0 μM) were added into corresponding wells in probe plates. The probe plate was first entered into the instrument to run the calibration procedure, and then changed to the cell plate for OCR detection. At the end of the assay, protein concentration of the cells were measured for data normalization.

### 4.15 Cell apoptosis analysis

In this study, the Annexin V-FITC/PI Cell Apoptosis Detection Kit (TransDetect, FA101) was utilized. Following trypsin digestion (without EDTA), cells were washed twice with PBS buffer and then double-stained with Annexin V-FITC and PI stains. Simultaneously, an equal number of unstained cells were used as a negative control to differentiate normal living cells. Subsequently, the samples were incubated at room temperature for 15 min in the dark and analyzed using a quantitative imaging flow cytometer (Amnis ImageStreamX MarkII, Millipore).

### 4.16 Mitochondria morphology observation

To observe mitochondrial morphology, the laser confocal microscope (Zeiss LSM 900, Germany) was used [64]. The cells were seeded on the 0.17 mm bottom thickness confocal dedicated dishes for overnight. Then cells were washed twice with PBS buffer (10 mM, pH 7.4) and then treated with 10 nM MitoTracker™ Red CMXRos (Invitrogen, M46752) in dark for 15 min. After that, cells were washed twice to remove excess dye. Lastly, the stained cells were suspended in 1 ml PBS and the red fluorescence was detected using a confocal microscope.

### 4.17 Transmission electron microscope

Cells were fixed with 2 % paraformaldehyde and 2.5 % glutaraldehyde at 4 °C for 1 h, then washed with PBS, fixed with 2 % osmium tetroxide for 1 h and washed. Subsequently, samples were dehydrated in a gradient ethanol series, replaced in acetone twice, infiltrated in resin-acetone mixtures at ratios of 1/4, 1/2, and 3/4 for 1 h each, and embedded in pure resin for 4 h. Subsequently, the samples were subjected to heat-induced curing, sectioning, and stained with 2 % uranyl acetate and 3 % lead citrate, followed by observation and analysis under a transmission electron microscope (ZEISS).

### 4.18 HS_n_H preparation

For HS_n_H preparation, 25.6 mg sulfur power, 44.8 mg NaHS, and 32 mg NaOH were added into 20 ml of oxygen-free deionized water under argon gas, after gentle mixing, the anaerobic bottle was sealed and incubated at 37 °C. After 2–3 days, the process continued until the sulfur power was completely dissolved. The concentration of HS_n_H was determined according to a previous report, with thiosulfate as the standard for calibration [65].

### 4.19 H&E staining

First, the fresh tissues were fixed with 4 % paraformaldehyde at 4 °C for overnight, and performed for paraffin embedding, then cut into 5 μm paraffin bands, and stained with hematoxylin/eosin to assess histology and morphology of tumors. The stained tissues were observed through an optical microscope (OLYMPUS DP80, Japan) and photographed (25.2 × ).

### 4.20 RNA extraction and qPCR

Total RNA from cultured cells was extracted with RNA Kit (Omega, R6834) following the manufacturer's protocol [66]. RNA sample (2 μg) was reversely transcribed into cDNA by a reverse transcription kit (Thermo Scientific, USA), primers used for qPCR were listed in Supplementary Table S3.

### 4.21 Enzymatic activity assay

To determine the effect of sulfane sulfur on the enzymatic activity of purified ALDOA, 1 mg/ml purified ALDOA were treated with different concentrations of sulfane sulfur for 10 min. The aldolase activity was measured using the Fructose-bisphosphate aldolase (FBA) Activity Assay Kit (Solarbio, BC2275). For crude enzymatic activity assay, tumor tissue blocks of same weight were ground with liquid nitrogen, then added to PBS buffer, shock and mixed, centrifuged for 10 min, the concentration was determined using the BCA assay, and crude enzymatic activity was detected using FBA Activity Assay Kit.

### 4.22 Western blotting analysis

Total protein was extracted by liquid nitrogen and quantified by BCA assay. Proteins were separated by SDS-PAGE and transferred to a PVDF membrane (Millipore), then the membranes were blocked with 5 % skimmed milk for 2 h. Then incubated using primary antibodies for overnight, followed by incubation with appropriate secondary antibodies (HRP-*anti*-rabbit IgG or HRP-*anti*-mouse IgG, 1:5000 dilution). An enhanced chemiluminescence was used to detect signals.

### 4.23 Targeted metabolomics analysis

The tumor tissues were quickly frozen in liquid nitrogen immediately after dissection, and then the tissues were cut on dry ice into an eppendorf tube (2 ml). The tissue sample was mixed with 200 μl H_2_O and five ceramic beads and was homogenized. After the homogenization, 800 μl methanol/acetonitrile (1:1, v/v) was added and the sample was centrifuged at 14,000 *g* for 20 min at 4 °C condition. The obtained supernatant was dried in a vacuum centrifuge and re-dissolved in 100 μl acetonitrile/water (1:1, v/v) solvent for LC-MS analysis.

Analyses were performed using an UHPLC (1290 Infinity LC, Agilent Technologies) coupled to a QTRAP MS (AB 6500+, AB Sciex). The analytes were separated on HILIC (Waters ACQUITY UPLC BEH Amide column, 2.1 mm × 100 mm, 1.7 μm) and C18 columns (Waters ACQUITY UPLC BEH C18–2.1 × 100 mm, 1.7 μm). For HILIC separation, the column temperature was 35 °C; and the injection volume was 2 μl. Mobile phase A: 90 % H_2_O + 2 mM ammonium formate +10 % acetonitrile, mobile phase B: 0.4 % formic acid in acetonitrile. A gradient (85 % B at 0–1 min, 80 % B at 3–4 min, 70 % B at 6 min, 50 % B at 10–15.5 min, 85 % Bat 15.6–23 min) was then initiated at a flow rate of 300 μl/min. For RPLC separation, the column temperature was set at 40 °C, and the injection volume was 2 μl. Mobile phase A: 5 mM ammonium acetate in water, mobile phase B: 99.5 % acetonitrile. A gradient (5 % B at 0 min, 60 % B at 5 min, 100 % B at 11–13 min, 5 % B at 13.1–16 min) was then initiated at a flow rate of 400 μl/min. The sample was placed at 4 °C during the whole analysis process.

6500 + QTRAP (AB SCIEX) was performed in positive and negative switch mode. The ESI positive/negative source conditions were as follows: source temperature: 580 °C; Ion Source Gas1 (GS1): 45; Ion Source Gas2 (GS2): 60; Curtain Gas (CUR): 35; Ion Spray Voltage (IS): +4500 V or - 4500 V in positive or negative modes, respectively. MRM method was used for mass spectrometry quantitative data acquisition. Metabolites with p-value*<*0.05 and fold change*>*1.5 were considered as significantly different.

### 4.24 Transcriptomic analysis

Transcriptomic analysis of the tumors was performed by Shanghai Applied Protein Technology Co., Ltd (Shanghai, China). Firstly, the total RNA was extracted and the RNA Integrity Number value met the standard required for preparing a cDNA library. Then the cDNA libraries were prepared, magnetic beads with Oligo (dT) were used to enrich mRNA and fragmented into small pieces using fragmentation buffer. The mRNA fragments were converted into the first strand of cDNA with random hexamers primers and Reverse Transcriptase (RNase H) using mRNA fragments as template, then the second strand of cDNA was synthesized followed by adding buffer, dNTPs, RNase H and DNA polymerase I. Then the double stranded cDNA was purified using AMPure XP system and then A-tailing and sequencing adapters were connected. PCR amplification was performed to obtain the final cDNA library. The library quality was assessed on Agilent Bioanalyzer 4150 system, the library was sequenced using Illumina NovaSeq 6000 and 150 bp paired-end reads were generated. The clean reads were separately aligned to the genome of Homo sapiens (Human) with orientation mode using HISAT2 software to obtain mapped reads.

### 4.25 Protein expression, purification

The ORF of aldolase A were ligated into pET-15b plasmid for expression, and transformed into *E. coli* BL21 (DE3). The positive transformed cells were selected by adding ampicillin antibiotics. *E. coli* cells containing aldolase plasmid were cultured in LB medium until OD_600 nm_ reached about 0.6 at 37 °C and then induced by a final concentration of 0.5 mM isopropyl b-d-1-thiogalactopyranoside (IPTG) at 16 °C for overnight. The cells were harvested by centrifugation and resuspended in buffer I (50 mM Na_2_HPO_4_/NaH_2_PO_4_, 250 mM NaCl, 20 mM imidazole, pH 8.0), then disrupted using a pressure cell homogenizer (SPCH-18) at 4 °C, the His-tagged protein was purified using Ni-NTA-Sefinose column (Cytiva, Sweden) following the manufacturer's instructions. Aldolase was concentrated using 30 K centrifugal concentrator and its concentration was determined using the BCA assay.

### 4.26 LC-MS/MS analysis of ALDOA protein

The analysis was performed following a previous report [67]. For DTT-treated, H_2_S-treated and H_2_S_n_ treated-ALDOA, freshly purified protein (<100 μg) were prepared for each group, and treated with 10-fold amounts of DTT (600 μM), H_2_S (600 μM) and H_2_S_n_ (600 μM) at room temperature for 1 h, respectively. Then the mixture were treated with denaturing buffer (8 M urea) containing 100 mM β-(4-hydroxyphenyl)ethyl iodoacetamide (HPE-IAM), the samples were centrifuged through Microcon YK-10 K ultrafiltration tubes at 13,000 *g* at 4 °C and resuspend in 25 mM NH_4_HCO_3_. Then the samples was transferred into new EP tubes and digested with trypsin (1:25, w/w) at 37 °C for 20 h. The digestion products were filtered by C_18_ Zip-Tip (Millipore) and dried in a vacuum concentrator. The obtained peptides were resuspended in 10 μl ddH_2_O.

LC-MS/MS data analysis was conducted following a previously reported protocol [68]. The Prominence nano-LC system (Shimadzu) equipped with a custom-made silica column (75 μm × 15 cm) packed with 3-μm Reprosil-Pur 120C18-AQ was used for the analysis. Positively electrospray ionization was performed, and the ions were scanned with an LTQ-Orbitrap Velos Pro CID mass spectrometer (Thermo Scientific); the data were analyzed using a data-dependent acquisition mode with Xcalib 2.2.0 software (Thermo Scientific). Full-scan MS spectra (from 400 to 1800 *m/z*) were detected and assessed with the Orbitrap at a resolution of 60,000 at 400 *m/z*.

### 4.27 Mitochondrial isolation

The mitochondrial extraction was performed following previous reports [69,70]. A total of 2 × 10^6^ cells were collected and resuspended in 1 ml of ice-cold mitochondrial isolation buffer (Beyotime Biotechnology, C3601). The resulting suspension was subsequently transferred to an appropriately sized homogenizer and homogenized 20 times. All procedures were conducted on ice to maintain low temperatures. The homogenate samples were then centrifuged at 600×*g* for 10 min at 4 °C. Following this, the supernatants were carefully collected into new 1.5 ml conical tubes and subjected to further centrifugation at 11,000×*g* for 10 min at 4 °C. Finally, the supernatants were discarded, and the pellets were washed twice and resuspended in mitochondria store buffer (Beyotime Biotechnology, C3609) for further analysis. Mitochondria concentration was determined using the BCA assay.

### 4.28 Measurement of ubiquinone (CoQ_10_) and ubiquinol (CoQ_10_H_2_) by UHPLC-MS/MS

The quantification of CoQ_10_ and CoQ_10_H_2_ were conducted according to previous reports [33,34]. Mitochondrial pellets were resuspended in 500 μl of ethanol and vortexed for 10 min at 4 °C. The suspension was then transferred to a new 2 ml tube, followed by the addition of 1 ml hexane and vortexing again for 10 min at 4 °C. Subsequently, the mixture was centrifuged at 16,000×*g* for 10 min at 4 °C, resulting in the formation of two distinct layers: hexane (upper layer) and ethanol (lower layer). The hexane layer was carefully collected and transferred to a new 1.5 ml tube, where it was dried using a Genevac miVac DNA Benchtop vacuum concentrator. Before UHPLC-MS analysis the dried metabolites were resuspended in 50 μl isopropanol and centrifuged at 12,000×*g* for 10 min.

UHPLC-MS/MS was performed on an Ultra-High Performance liquid chromatography system (SCIEX, ExionLC, UHPLC) coupled with a triple quadrupole mass spectrometer (SCIEX Triple Quad 5500+ QTrap Ready). Chromatographic separation of CoQ_10_ and CoQ_10_H_2_ was achieved on an Agilent Shim-pack GIST-C18 column (4.6 × 250 mm, 5 μm). The mobile phase consisted of solvent A (isopropanol) and solvent B (acetonitrile/H_2_O, 7:1, containing 10 mM ammonium acetate), delivered at a flow rate of 0.4 ml/min. The percentage of solvent A was increased linearly from 35 to 85 % over 6 min. The mass spectrometer was operated in positive electrospray ionization (ESI) utilizing the multiple reaction (MRM) mode with a dwell time of 200 ms per transition. The contents of CoQ_10_ and CoQ_10_H_2_ were quantified by integrating the peak areas using TraceFinder 4.1 software (ThermoFisher). The molecular masses of proton adduct used for detection were *m/z* 863.7 for CoQ_10_ and *m/z* 865.7 for CoQ_10_H_2_.

### 4.29 Quantification and statistical analysis

The data displayed are the mean ± SD from three samples, as quantified by ImageJ (National Institutes of Health, USA) in the analysis of western blots. Data analysis was conducted utilizing the GraphPad Prism 9.0 (GraphPad Software, USA). The results are articulated as the mean values ± SD. *p*-values were ascertained employing two-tailed Student's *t*-tests or Mann–Whitney *U* tests for statistical significance [41], with statistical significance determined at the threshold of *p* < 0.05. The corresponding *p*-values are delineated within the illustrative figures, and the numerical details pertaining to replicates, independent samples, and animal subjects are specified in the associated figure legends.

## CRediT authorship contribution statement

**Ting Lu:** Writing – original draft, Validation, Investigation, Formal analysis. **Qingda Wang:** Writing – original draft, Investigation. **Yuping Xin:** Writing – original draft, Investigation. **Xiaohua Wu:** Writing – original draft, Investigation. **Yang Wang:** Writing – original draft, Investigation. **Yongzhen Xia:** Writing – original draft, Formal analysis. **Luying Xun:** Writing – review & editing, Supervision, Resources, Project administration, Conceptualization. **Huaiwei Liu:** Writing – review & editing, Supervision, Funding acquisition, Conceptualization.

## Funding

This work was supported by grants from the 10.13039/501100001809National Natural Science Foundation of China (92351302, 32300076), and the 10.13039/501100007129Natural Science Foundation of Shandong Province, China (ZR2023QC185).

## Declaration of competing interest

The authors declare that they have no known competing financial interests or personal relationships that could have appeared to influence the work reported in this paper.

## Data Availability

The transcriptomic raw data have been deposited at SRA in National Center for Biotechnology Information (NCBI) and are publicly available as of the date of publication. Accession numbers is PRJNA1125503.
