# Supplementary material for: Knockout of the sulfide: quinone oxidoreductase SQR reduces growth of HCT116 tumor xenograft
Source: Redox Biol. 2025 Apr 24;83:103650. doi: 10.1016/j.redox.2025.103650 (PMC12433914; doi:10.1016/j.redox.2025.103650)
Supplement: Multimedia component 1 [file mmc1.docx]

**Supporting material**

Knockout of the sulfide: quinone oxidoreductase SQR reduces growth of HCT116 tumor xenograft

Ting Lu^1,2#^, Qingda Wang^2#^, Yuping Xin^2^, Xiaohua Wu^2^, Yongzhen Xia^2^, Luying Xun^2,3^, Huaiwei Liu^2^*

^1^School of Health and Life Sciences, University of Health and Rehabilitation Sciences Qingdao Hospital (Qingdao Municipal Hospital), University of Health and Rehabilitation Sciences, Qingdao 266071, People’s Republic of China.

^2^State Key Laboratory of Microbial Technology, Shandong University, Qingdao, 266200, People’s Republic of China.

^3^School of Molecular Biosciences, Washington State University, Pullman, WA, 991647520, USA.

*Correspondence: [liuhuaiwei@sdu.edu.cn](mailto:liuhuaiwei@sdu.edu.cn)

**^#^**These authors contributed equally to this work.

Content:

Figure S1-Figure S11

Table S1-Table S3

**
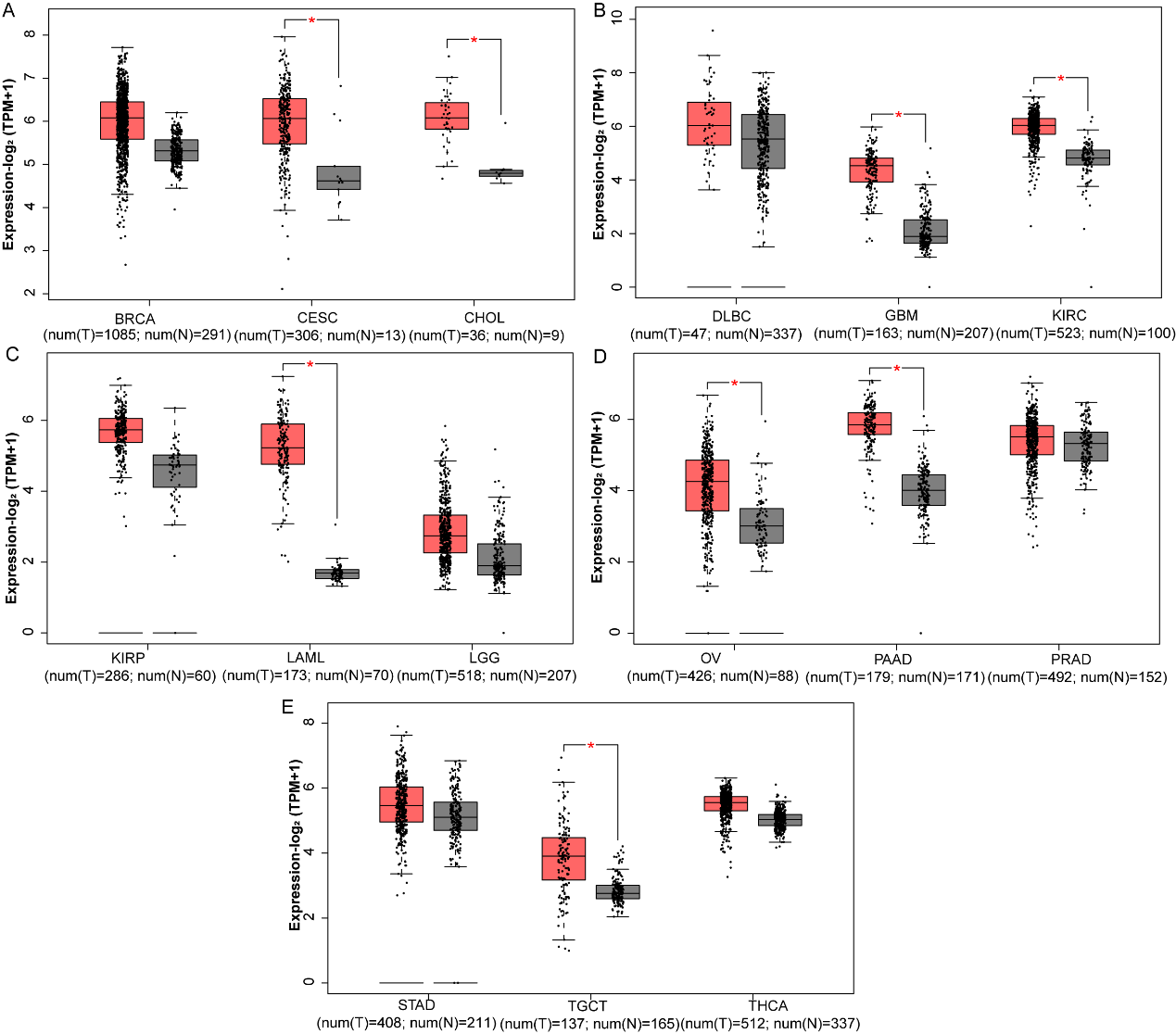
**

**Figure S1. Expression analysis of *sqr* in different clinical tumor samples.** (A-E) Expression levels of *sqr* in different clinical tumor samples were analyzed by GEPIA database. T and N represent tumor tissue samples and corresponding normal tissue samples, respectively, with the abbreviations for tumor types defined as follows. BRCA: Breast invasive carcinoma; CESC: Cervical squamous cell carcinoma and endocervical adenocarcinoma; CHOL: Cholangiocarcinoma; DLBC: Diffuse large B-cell lymphoma; GBM: Glioblastoma multiforme; KIRC: Kidney renal clear cell carcinoma; KIRP: Kidney renal papillary cell carcinoma; LAML: Acute myeloid leukemia; LGG: Brain lower grade glioma; OV: Ovarian serous cystadenocarcinoma; PAAD: Pancreatic adenocarcinoma; PRAD: Prostate adenocarcinoma; STAD: Stomach adenocarcinoma; TGCT: Testicular germ cell tumors; THCA: Thyroid carcinoma. The two-tailed unpaired Student’s *t*-tests were performed for (A), (B), (C), (D) and (E). **p*<0.05.

**
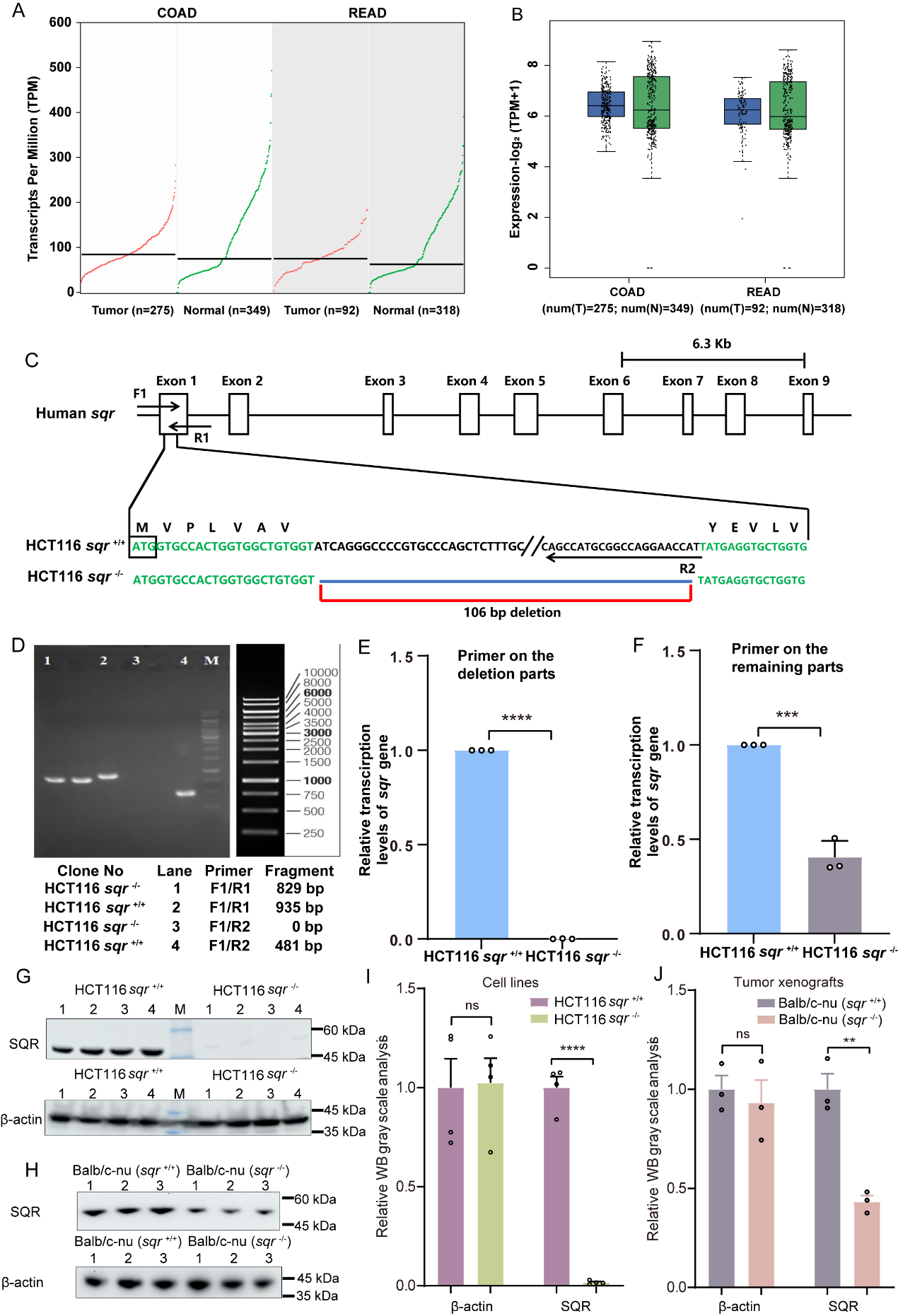
**

**Figure S2. Expression analysis of *sqr* in CRC samples and construction of *sqr*^-/-^ cell line. Related to Figure 1 and 2.** (A-B) The transcripts million (TPM) level (A) and exponential expression-log_2_ (TPM+1) level (B) of SQR in two types of cancer types. Data were generated by GEIPA analysis. (C) The location of deleted DNA sequence in *sqr* gene. A 106-bp fragment in exon1 was targeted. F1, R1, and R2 are primers used for verifying the deletion. (D) PCR verification of the deleted sequence in *sqr*^-/-^ cell using F1, R1, and R2 primers. (E) RT-qPCR analysis of the relative transcription level of *sqr* gene using a pair of primers inside the deleted sequence (*n* = 3 each). (F) RT-qPCR analysis of the relative transcription levels of *sqr* gene using a pair of primers located in the undeleted sequence (*n* = 3 each). (G and H) SQR protein level in cell lines and tumors were analyzed by western blotting. β-actin protein was used as an internal control. (I and J) The gray values of the western blotting bands in cell lines (n = 4 each) and tumors (n = 3 each) were analyzed by Image J software. WB grayscale is the relative value of samples on the same film. Data were from three independent repeats and shown as average ± SD. A two-tailed paired Student’s *t*-tests was performed for (E-F, I-J). ns: no significance, **p*<0.05, ***p*<0.01, ****p*<0.001, *****p*<0.0001.

**
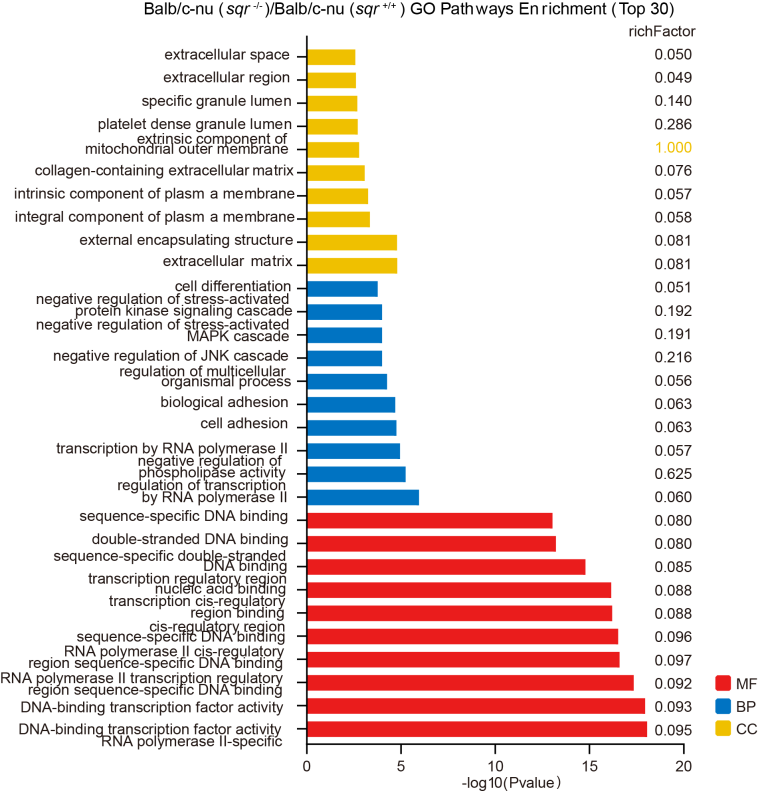
**

**Figure S3. GO analysis of differentially expressed genes (DEGs). Related to Figure 4.** Top 10 terms of DEGs for MF, BP and CC, respectively. The numbers in right indicate rich factors. "Rich factor" refers to the ratio of the number of detected differential metabolites to the total metabolites of the corresponding pathway in the KEGG database. MF stands for molecular function, BP stands for biological process, and CC stands for cellular component.

**
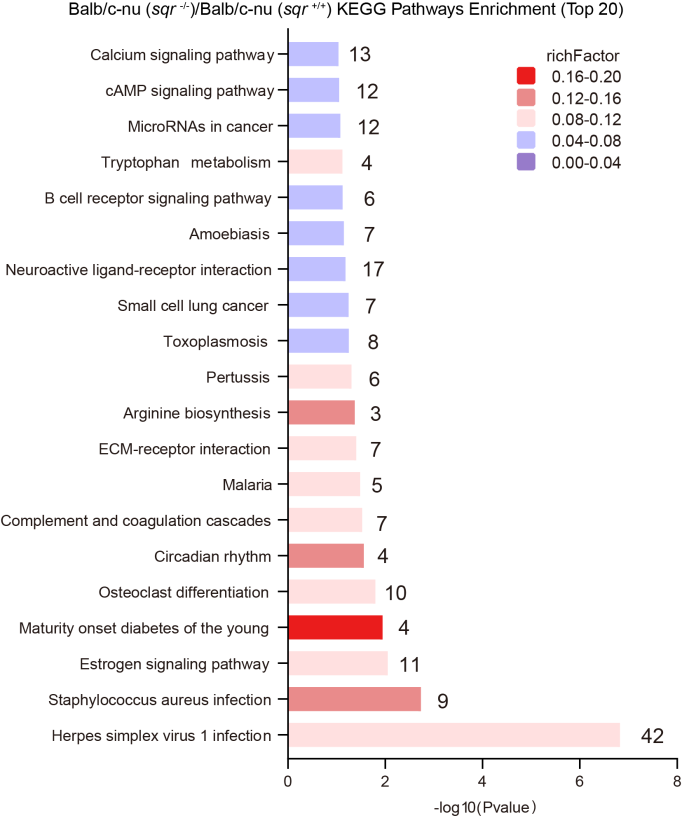
**

**Figure S4. KEGG analysis of differentially expressed genes.** **Related to Figure 4.** Top 20 KEGG pathways of DEGs. The color indicates rich factor and the number showes the count of DEGs enriched. "Rich factor" means the ratio of the number of detected differential metabolites to the total metabolites of the corresponding pathway in the KEGG database.

**
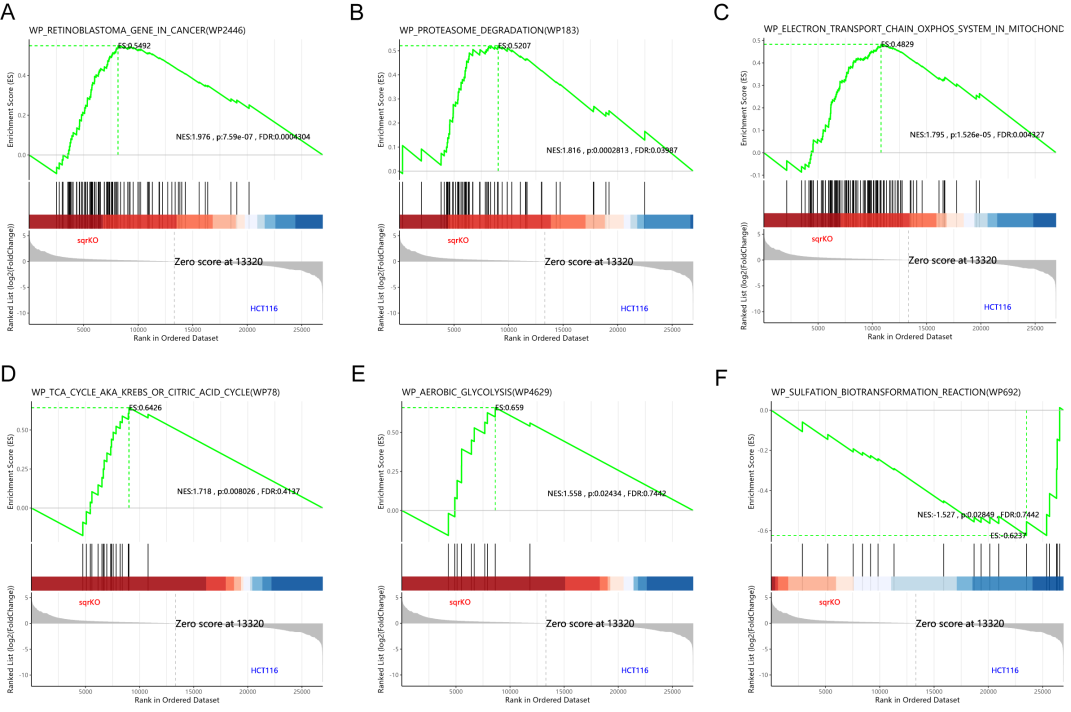
**

**Figure S5. GSEA analysis of the WiKipathway dataset****.** **Related to Figure 4.** (A-D) GSEA gene expression profiles of retinoblastoma gene in cancer (No.1 ranking), proteasome degradation (No.2 ranking), electron transport chain oxidative phosphorylation system in mitochondria (No.3 ranking), TCA cycle (No.4 ranking). (E-F) GSEA gene expression profiles of aerobic glycolysis (No.11 ranking) and sulfation biotransformation reaction (No.15 ranking).


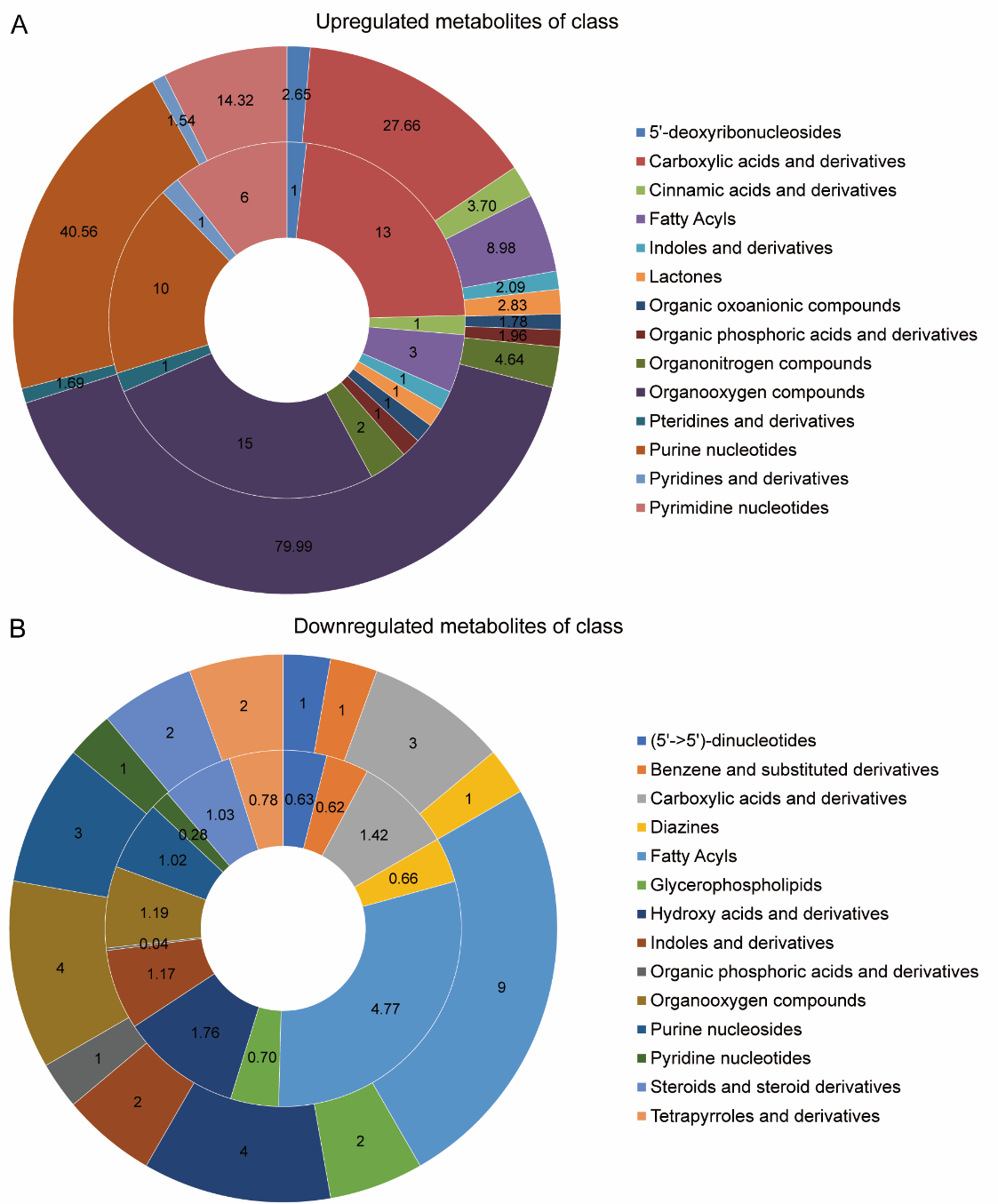


**Figure S6. Content changed metabolites identified by targeted metabolomics. Related to Figure 5.** (A) Class of content increased metabolites. Inner ring represents Balb/c-nu (*sqr*^+/+^) groups, outer ring represents Balb/c-nu (*sqr*^-/-^) groups, each class of Balb/c-nu (*sqr*^+/+^) groups metabolite is set to 1. Balb/c-nu (*sqr*^-/-^) groups were the fold values compared with Balb/c-nu (*sqr*^+/+^) groups. The area of the inner ring represents the number of metabolites of the major class, while the area of the outer ring is the rise multiple of the corresponding inner ring metabolites. (B) Class of content decreased metabolites. Outer ring represents Balb/c-nu (*sqr*^+/+^) groups, inner ring represents Balb/c-nu (*sqr*^-/-^) groups, each class of Balb/c-nu (*sqr*^+/+^) groups metabolite is set to 1. Balb/c-nu (*sqr*^-/-^) groups were the fold values compared with Balb/c-nu (*sqr*^+/+^) groups. The area of the outer ring represents the number of metabolites of the large class, while the area of the inner ring is the decline multiple of the corresponding outer ring metabolites. The names of metabolites and their corresponding classifications are shown in Table S2.


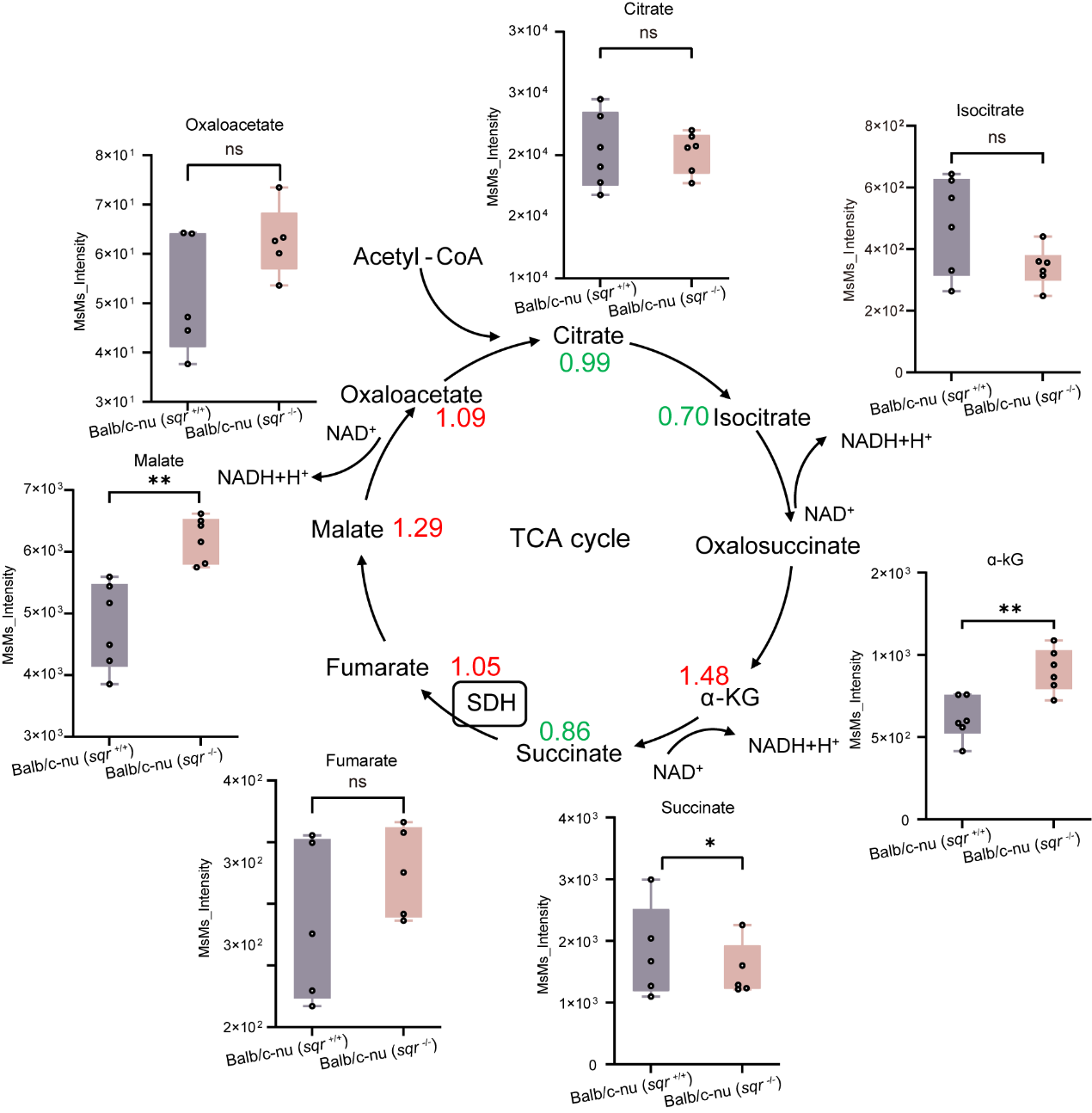


**Figure S7. The content change of metabolites in TCA cycle. Related to Figure 5 and 6.** There was no significant influence on metabolites of TCA cycle except α-ketoglutaric acid. The numbers next to the metabolites represent the ratio of Balb/c-nu (*sqr*^-/-^)/Balb/c-nu (*sqr*^+/+^). Data were from six independent repeats and shown as average ± SD. With the exception of the oxaloacetate n value of each 5, the remaining n values are each 6. Student’s t-test was performed for comparison. **p*<0.05, ***p*<0.01, ****p*<0.001, ns: no significant difference.

**
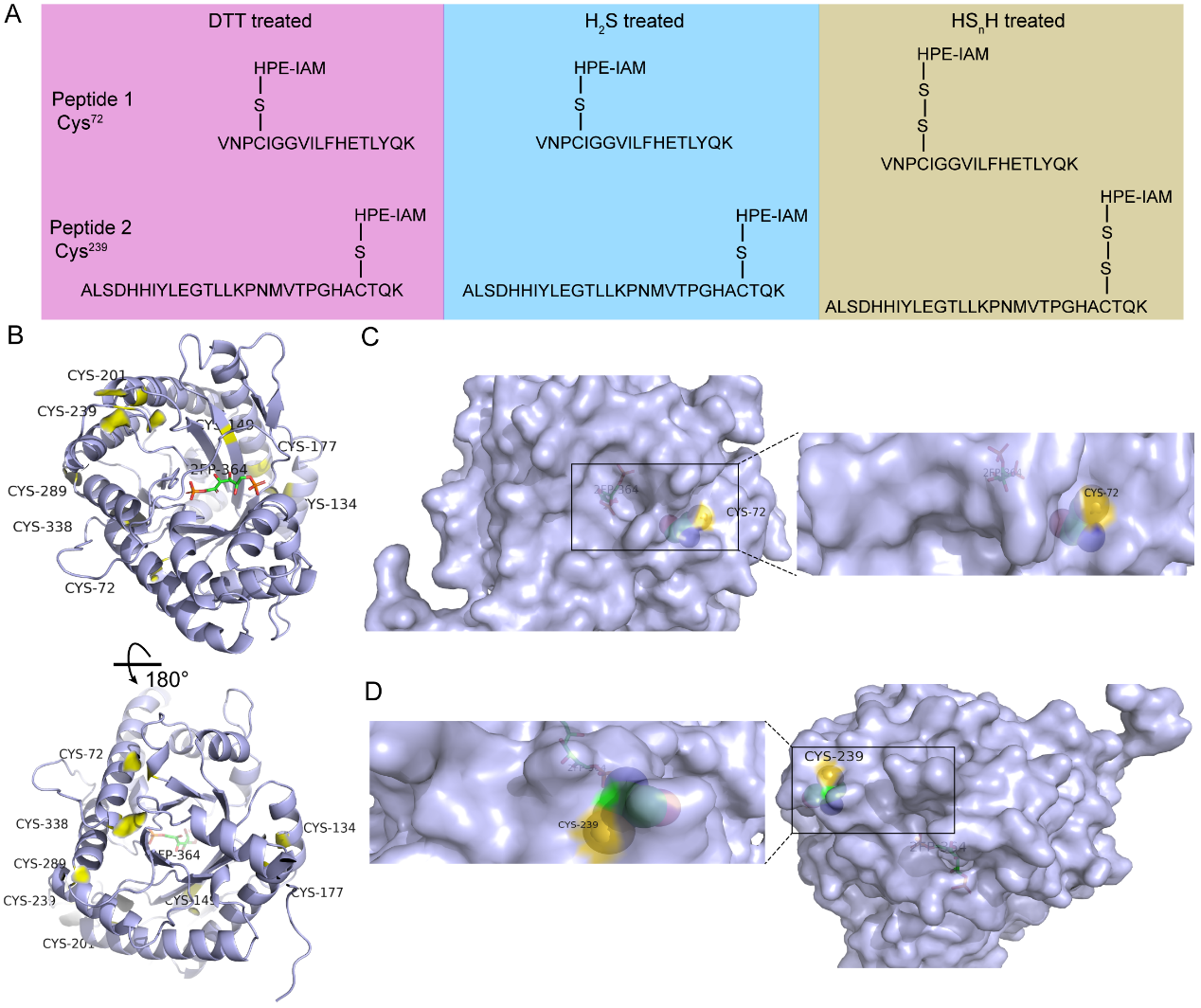
**

**Figure S8. LC-MS/MS and structure analysis of ALDOA.** (A) DTT, H_2_S and HS_n_H-treated ALDOA. MS^2^ data of specific peptides are provided in Figure S8 and S9. β-(4-hydroxyphenyl)ethyl iodoacetamide (HPE-IAM) was utilized as the thiol labeling reagents. (B) The structural view of 8 cysteines in ALDOA (PDB: 4ald) was displayed by pymol, and the structure was flipped 180 degrees. The protein backbone is shown in light blue, cysteines are shown in yellow, and the fructose 1,6-bisphosphate (2FB) is shown in green. (C and D) Cys^72^ and Cys^239^ are located on the surface of the ALDOA protein.


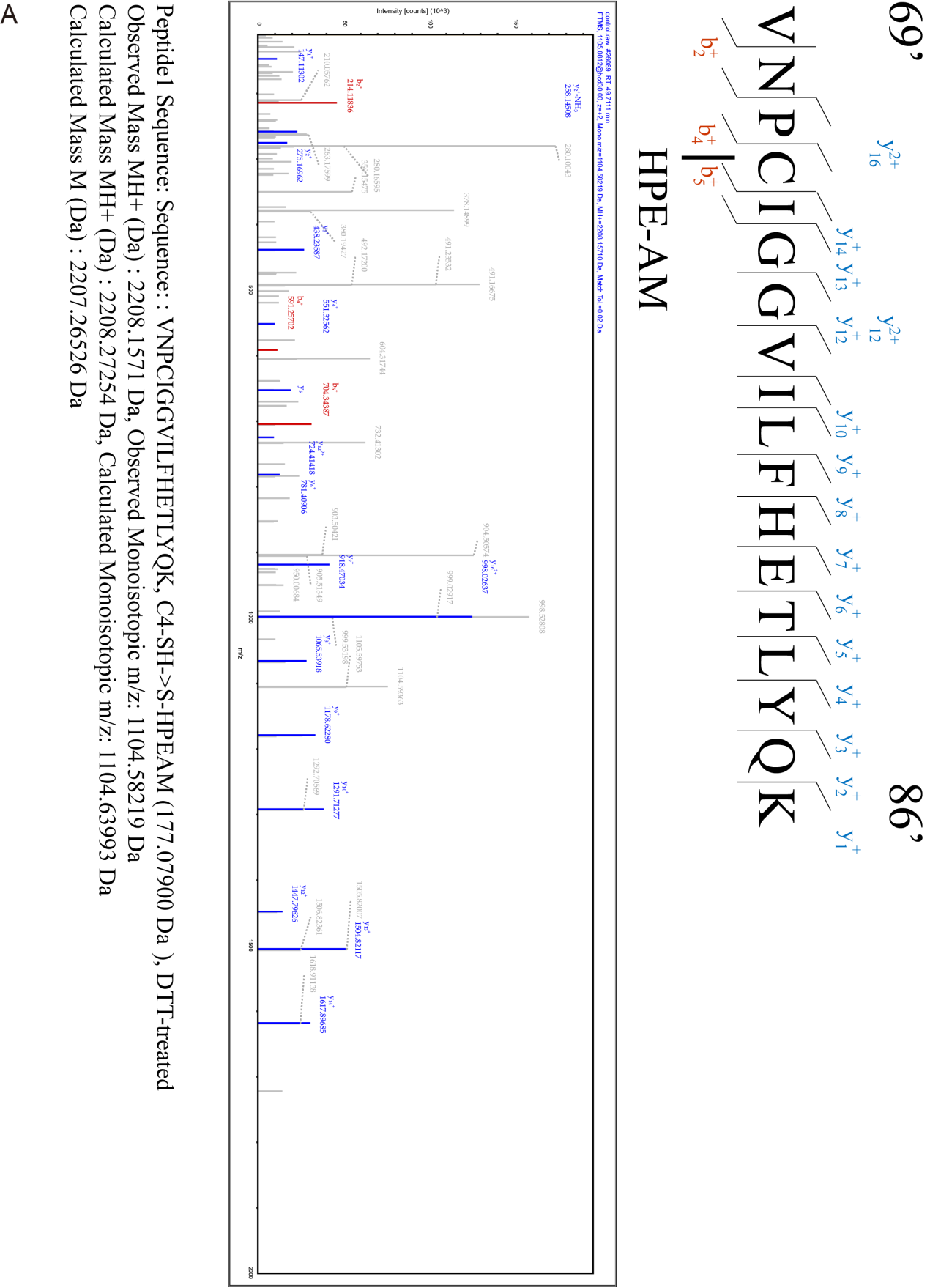


**Figure S9A. MS^2^ data of peptide 1, which was from DTT-treated ALDOA.**


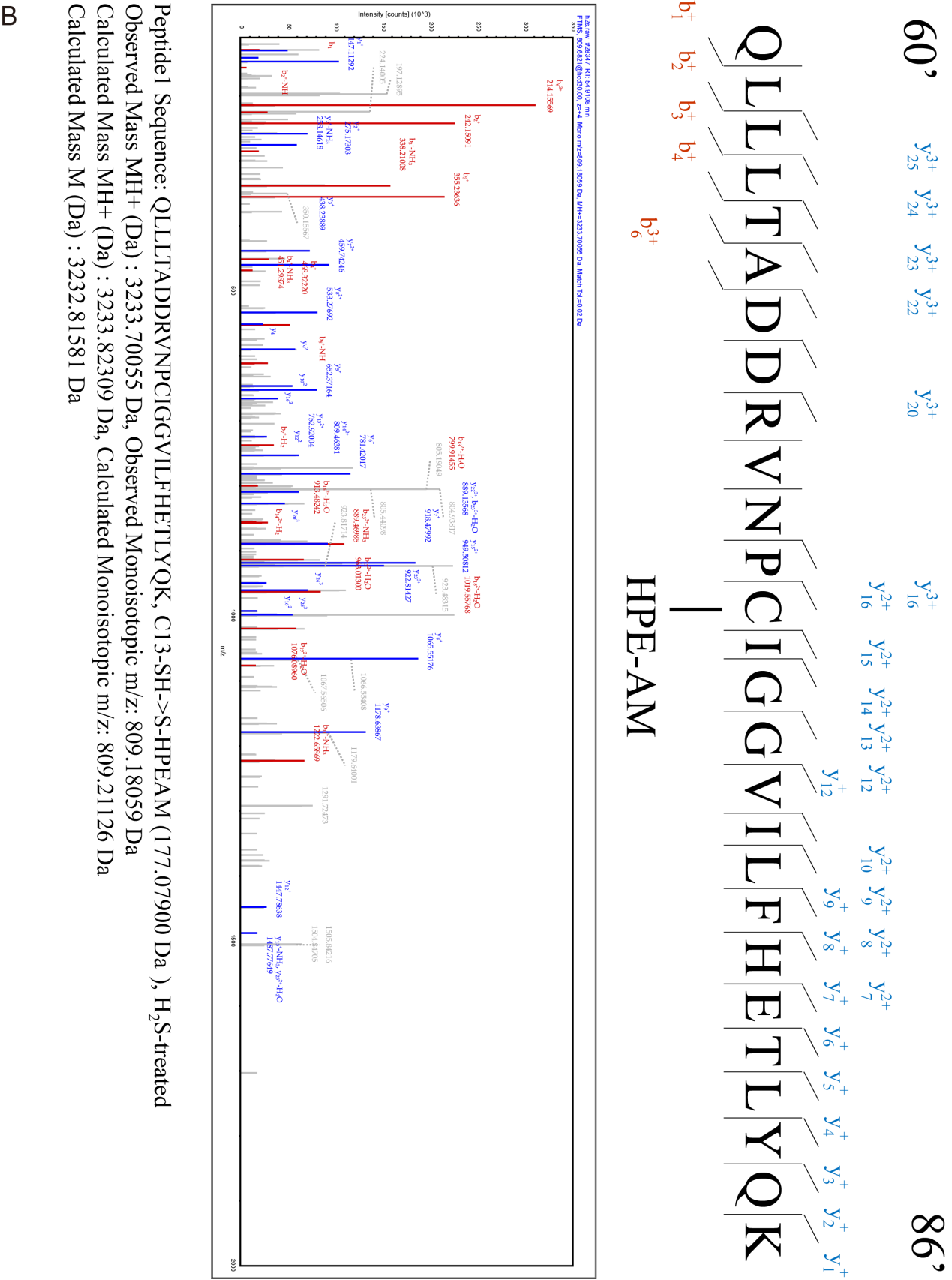


**Figure S9B. MS^2^ data of peptide 1, which was from H_2_S-treated ALDOA.**


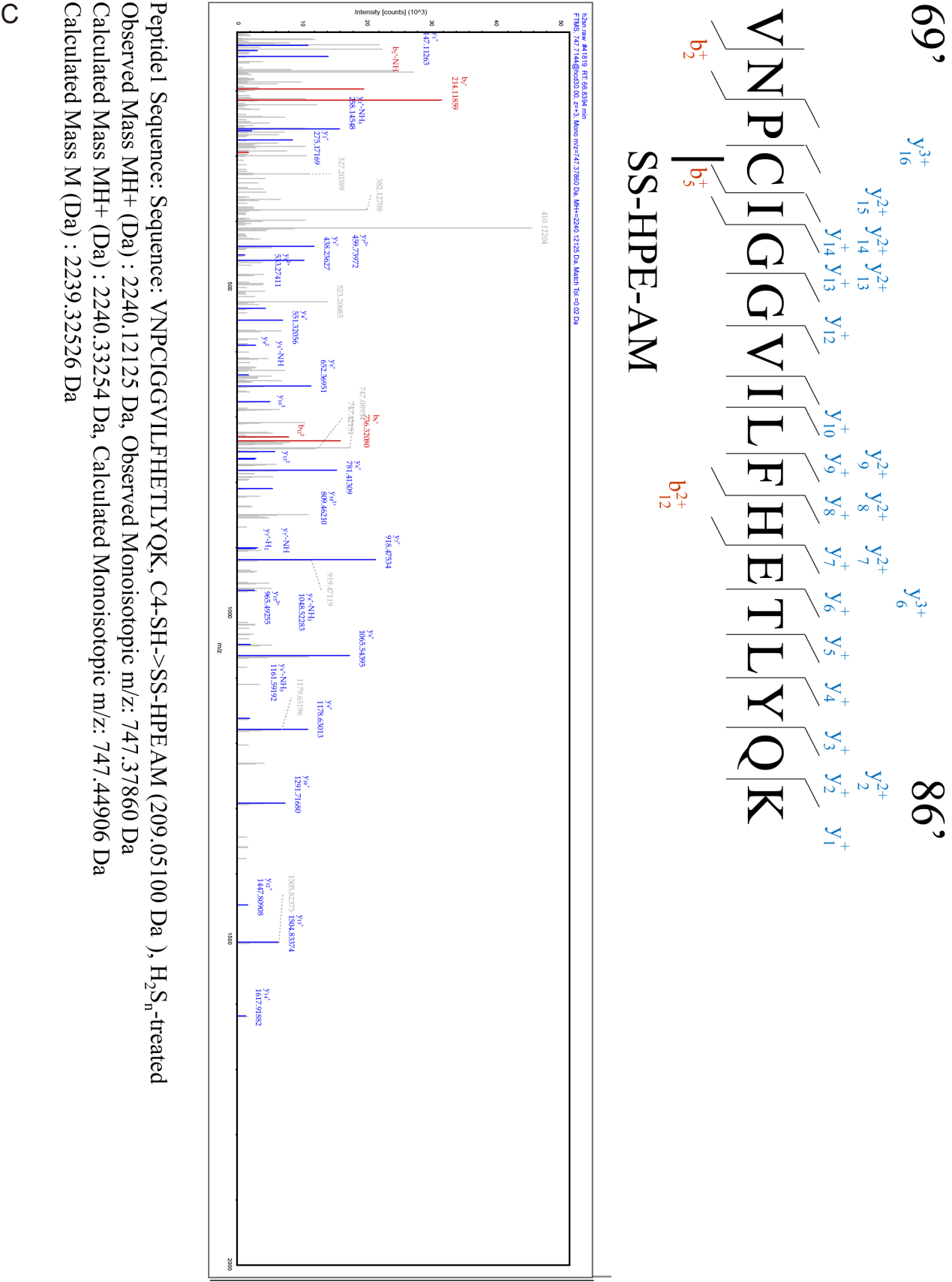


**Figure S9C. MS^2^ data of peptide 1, which was from H_2_S_n_-treated ALDOA.**


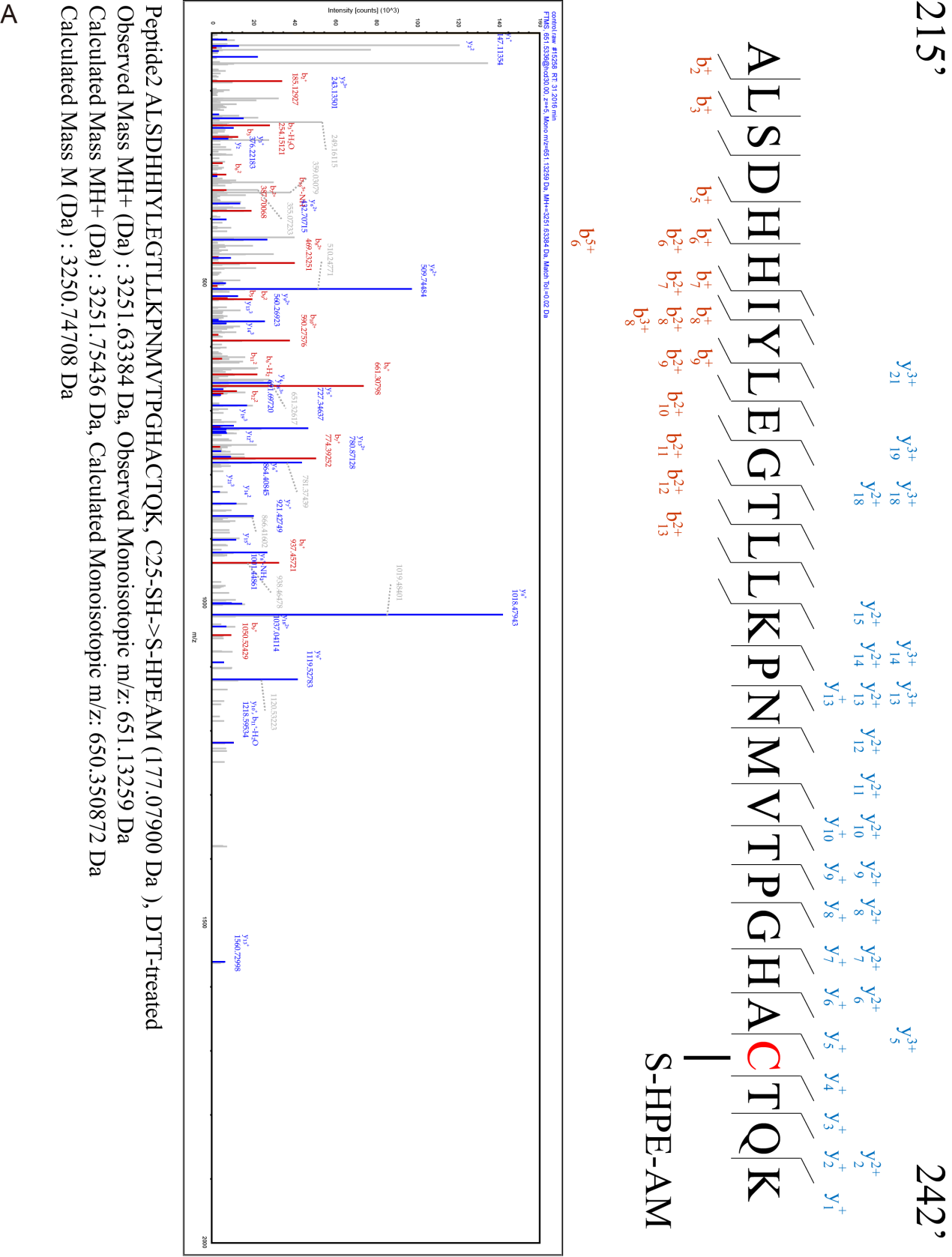


**Figure S10A. MS^2^ data of peptide 2, which was from DTT-treated ALDOA.**


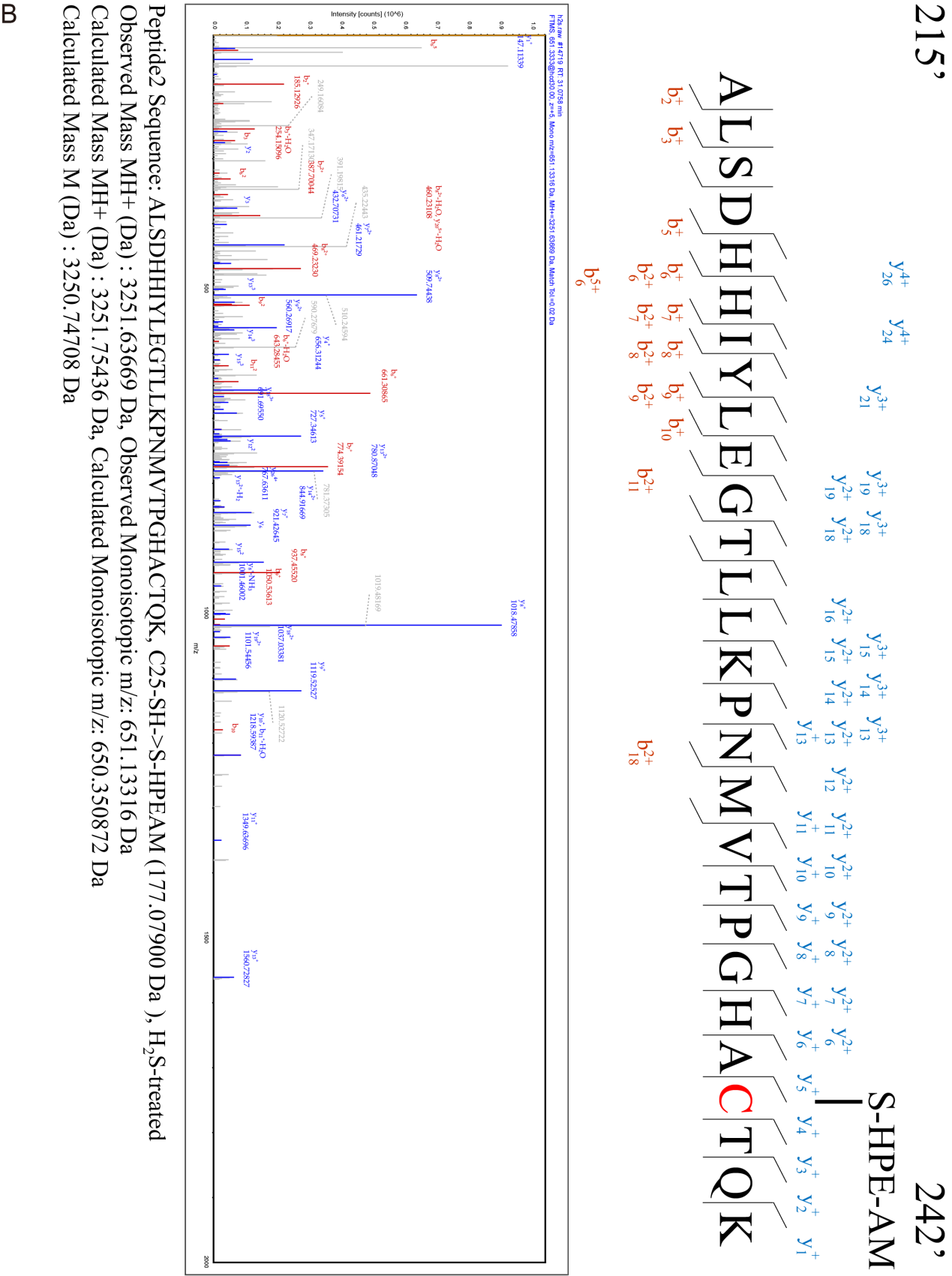


**Figure S10B. MS^2^ data of peptide 2, which was from H_2_S-treated ALDOA.**


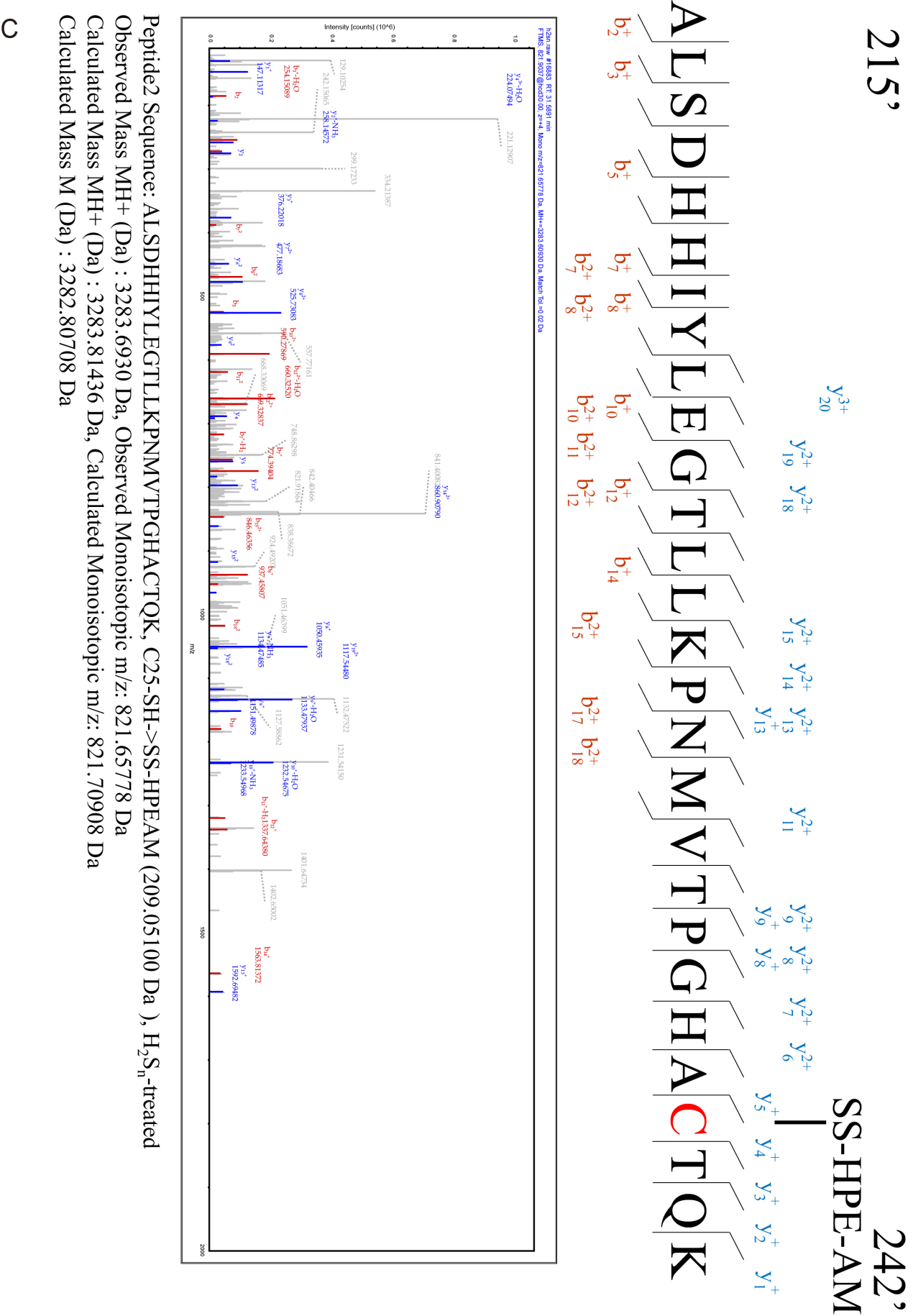


**Figure S10C. MS^2^ data of peptide 2, which was from H_2_S_n_-treated ALDOA.**

**
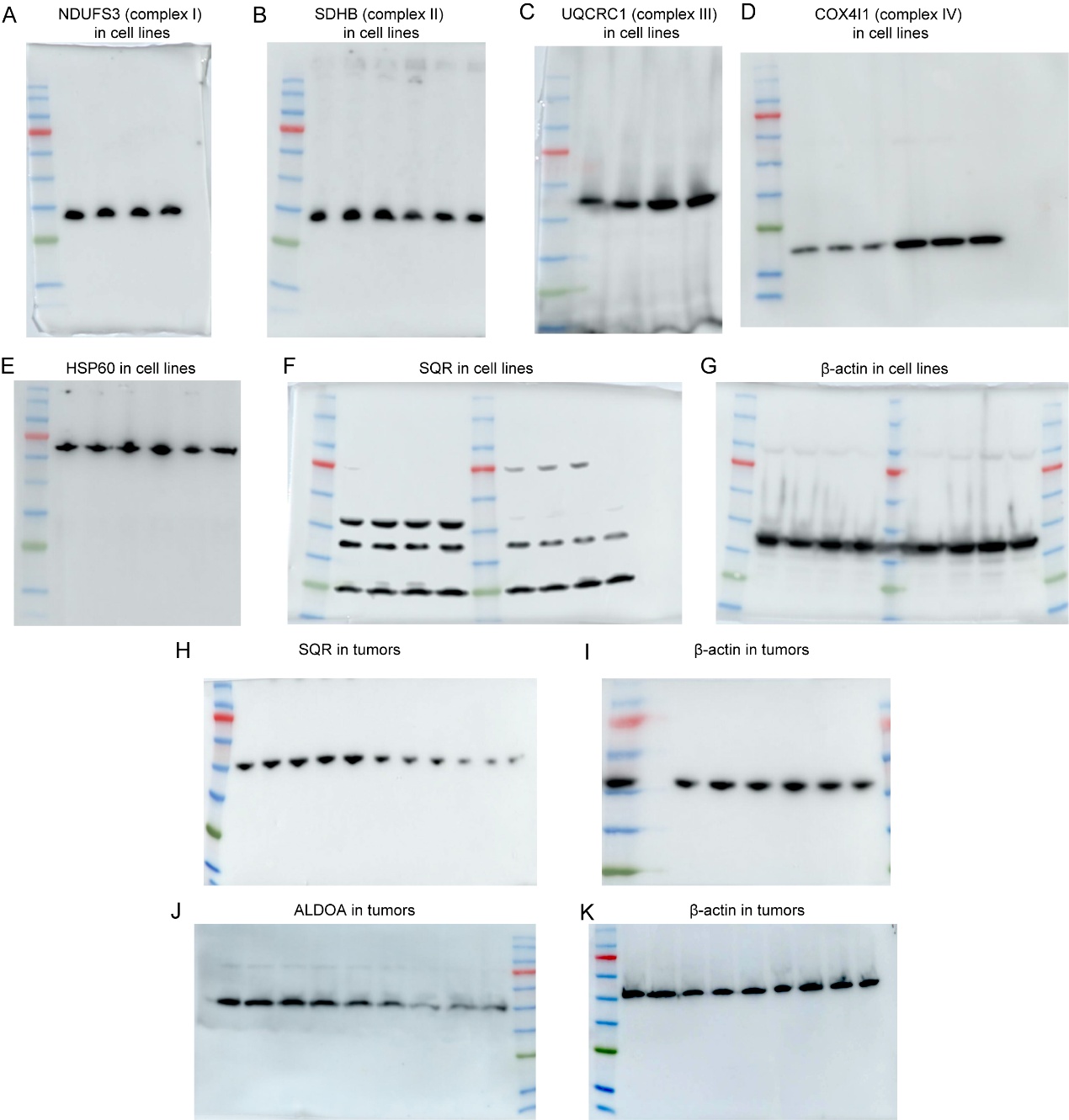
**

**Figure S11. The complete western blots of this study are presented.** Panels A-E of Figure S11 correspond sequentially to Figure 3C in the main text. Panels F-G of Figure S11 correspond sequentially to Figure S2G in the supplementary material. Panels H-I of Figure S11 correspond sequentially to Figure S2H in the supplementary material. Panels J-K of Figure S11 correspond sequentially to Figure 8G in the main text.

**Table S1. Detailed information on glycolytic gene abbreviations. Related to Figure 4.**

| Abbreviation | Gene function and coding protein |
| --- | --- |
| *gck* | glucokinase, GCK |
| *adpgk* | ADP dependent glucokinase, ADPGK |
| *hk1* | hexokinase 1, HK1 |
| *hkdc1* | hexokinase domain containing 1, HKDC1 |
| *hk2* | hexokinase 2, HK2 |
| *hk3* | hexokinase 3, HK3 |
| *galm* | galactose mutarotase, GALM |
| *g6pc1* | glucose-6-phosphatase catalytic subunit 1, G6PC1 |
| *g6pc2* | glucose-6-phosphatase catalytic subunit 2, G6PC2 |
| *g6pc3* | glucose-6-phosphatase catalytic subunit 3, G6PC3 |
| *gpi* | glucose-6-phosphate isomerase, GPI |
| *pfkl* | phosphofructokinase, liver type, PFKL |
| *pfkm* | phosphofructokinase, muscle, PFKM |
| *pfkp* | phosphofructokinase, platelet, PFKP |
| *fbp1* | fructose-bisphosphatase 1, FBP1 |
| *fbp2* | fructose-bisphosphatase 2, FBP2 |
| *aldoa* | aldolase, fructose-bisphosphate A, ALDOA |
| *aldob* | aldolase, fructose-bisphosphate B, ALDOB |
| *aldoc* | aldolase, fructose-bisphosphate C, ALDOC |
| *tpi1* | triosephosphate isomerase 1, TPI1 |
| *gapdh* | glyceraldehyde-3-phosphate dehydrogenase, GAPDH |
| *gapdhs* | glyceraldehyde-3-phosphate dehydrogenase, spermatogenic, GAPDHS |
| *pgk1* | phosphoglycerate kinase 1, PGK1 |
| *pgk2* | phosphoglycerate kinase 2, PGK2 |
| *bpgm* | bisphosphoglycerate mutase, BPGM |
| *minpp1* | multiple inositol-polyphosphate phosphatase 1, MINPP1 |
| *pgam4* | phosphoglycerate mutase family member 4, PGAM4 |
| *pgam1* | phosphoglycerate mutase 1, PGAM1 |
| *eno1* | enolase 1, ENO1 |
| *eno2* | enolase 2, ENO2 |
| *eno3* | enolase 3, ENO3 |
| *eno4* | enolase 4, ENO4 |

**Table S2. Metabolites name and their classification. Related to Figure 5.**

| **Name** | **class** |
| --- | --- |
| **Downregulated metabolites** | |
| Phosphoenolpyruvic acid | Organic phosphoric acids and derivatives |
| 2-Phosphoglyceric acid | Organooxygen compounds |
| 3-Phosphoglycerate | Organooxygen compounds |
| Nicotinamide ribotide | Pyridine nucleotides |
| 1-Methyladenosine | Purine nucleosides |
| N6-methyladenosine | Purine nucleosides |
| Cystine | Carboxylic acids and derivatives |
| Protoporphyrin | Tetrapyrroles and derivatives |
| Beta-Glycerophosphoric acid | Glycerophospholipids |
| 11Z-Eicosenoic Acid | Fatty Acyls |
| Glycerol 3-phosphate | Glycerophospholipids |
| Dodecanoylcarnitine | Fatty Acyls |
| Cytidine triphosphate | Organooxygen compounds |
| Alpha-Hydroxyisobutyric acid | Hydroxy acids and derivatives |
| Deoxyguanosine | Purine nucleosides |
| Hydroxypyruvic acid | Hydroxy acids and derivatives |
| 2-Hydroxybutyric acid | Hydroxy acids and derivatives |
| 3-Hydroxybutyric acid | Hydroxy acids and derivatives |
| Biliverdin | Tetrapyrroles and derivatives |
| 2-Hydroxycaproic acid | Fatty Acyls |
| Cystathionine | Carboxylic acids and derivatives |
| Myristelaidic acid | Fatty Acyls |
| Cholesterol sulfate | Steroids and steroid derivatives |
| 2-Hydroxy-3-methylbutyric acid | Fatty Acyls |
| Cortisol | Steroids and steroid derivatives |
| Indole-3-lactic Acid | Indoles and derivatives |
| Glyceric acid | Organooxygen compounds |
| 3-Indolepropionic acid | Indoles and derivatives |
| Myristoleic acid | Fatty Acyls |
| Dihydrofolic acid | Carboxylic acids and derivatives |
| 3-Aminosalicylic acid | Benzene and substituted derivatives |
| 1,4-Dihydronicotinamide adenine dinucleotide (NADH) | (5'->5')-dinucleotides |
| Palmitoylcarnitine | Fatty Acyls |
| Palmitoleic Acid | Fatty Acyls |
| Thiamine monophosphate | Diazines |
| Palmitelaidic acid | Fatty Acyls |
| **Upregulated metabolites** | |
| Methionine | Carboxylic acids and derivatives |
| Adenosine-3′,5′-cyclic monophosphate (cAMP) | Purine nucleotides |
| Nicotinamide | Pyridines and derivatives |
| Asparagine | Carboxylic acids and derivatives |
| N-Acetylmethionine | Carboxylic acids and derivatives |
| 5-Hydroxylysine | Carboxylic acids and derivatives |
| Dihydrobiopterin | Pteridines and derivatives |
| Uridine diphosphate-N-acetylglucosamine | Pyrimidine nucleotides |
| Glutaric acid | Carboxylic acids and derivatives |
| Methylsuccinic acid | Fatty Acyls |
| Deoxycytidine-diphosphate | Organic oxoanionic compounds |
| Uridine 5'-diphosphate (UDP) | Pyrimidine nucleotides |
| Uridine diphosphate-N-acetylgalactosamine | Pyrimidine nucleotides |
| Glutathione Reduced | Carboxylic acids and derivatives |
| Aminoadipic acid | Carboxylic acids and derivatives |
| Galacturonic acid | Organooxygen compounds |
| Guanidoacetic acid | Carboxylic acids and derivatives |
| Glucuronic acid | Organooxygen compounds |
| Histamine | Organonitrogen compounds |
| O-Phosphoethanolamine | Organic phosphoric acids and derivatives |
| myo-Inositol | Organooxygen compounds |
| D-Ribose 5-phosphate | Organooxygen compounds |
| Serotonin | Indoles and derivatives |
| Cysteinylglycine | Carboxylic acids and derivatives |
| Uridine diphosphategalactose (UDP-galactose) | Pyrimidine nucleotides |
| Gamma-Aminobutyric acid | Carboxylic acids and derivatives |
| Uridine diphosphate glucose (UDP-glucose) | Pyrimidine nucleotides |
| Trehalose | Organooxygen compounds |
| Adenosine diphosphate ribose (ADP-ribose) | Purine nucleotides |
| Glucosamine 6-sulfate | Organooxygen compounds |
| S-Adenosylmethionine | 5'-deoxyribonucleosides |
| Spermidine | Organonitrogen compounds |
| Uridine diphosphate glucuronic acid | Lactones |
| Ophthalmic acid | Carboxylic acids and derivatives |
| Guanosine diphosphate (GDP) | Purine nucleotides |
| Inosine-monophosphate (IMP) | Purine nucleotides |
| Guanosine diphosphate mannose (GDP-mannose) | Purine nucleotides |
| Heneicosanoic acid | Fatty Acyls |
| Gluconic acid | Organooxygen compounds |
| Leucyl-Glycine | Carboxylic acids and derivatives |
| 6-Phosphogluconic acid | Organooxygen compounds |
| Glycylleucine | Carboxylic acids and derivatives |
| Inosine 5'-diphosphate (IDP) | Purine nucleotides |
| Adenosine-5′-diphosphate (ADP) | Purine nucleotides |
| 2'-Deoxyguanosine 5'-diphosphate (dGDP) | Cinnamic acids and derivatives |
| Glucose 1-phosphate | Organooxygen compounds |
| Erucic Acid | Fatty Acyls |
| Guanosine monophosphate (GMP) | Purine nucleotides |
| Glucosamine 6-phosphate | Organooxygen compounds |
| Uridine 5'-monophosphate (UMP) | Pyrimidine nucleotides |
| Adenosine monophosphate (AMP) | Purine nucleotides |
| ADP-glucose | Purine nucleotides |
| Glucose 6-phosphate (G6P) | Organooxygen compounds |
| Fructose 1,6-bisphosphate (F1,6BP) | Organooxygen compounds |
| Galactose 1-phosphate | Organooxygen compounds |
| Mannose 6-phosphate | Organooxygen compounds |
| Fructose 6-phosphate | Organooxygen compounds |

**Table S3. Primers (5′→3′) used in this study. Related to STAR Methods.**

| Primer | Sequence (5'-3') | Function |
| --- | --- | --- |
| Sqr KO-F1 | GATGGTGCCCTAGATCTTACG | Sequencing verification of *sqr*^-/-^ knockout |
| Sqr KO-R1 | TGGCCTTGCCCATAGCTTGG |  |
| Sqr KO-R2 | ATGGTTCCTGGCCGCATGGCT |  |
| *Sqr inner* RT-F | CTGGTGGCTGTGGTATCAGG | RT-qPCR verification of *sqr*^-/-^ knockout |
| *Sqr* inner RT-R | ATAATGGTTCCTGGCCGCAT |  |
| *Sqr* RT3-F | TTATGAGGTGCTGGTGCTGG | RT-qPCR verification of *sqr*^-/-^ knockout |
| *Sqr* RT3-F | CCACATTCTCTGCACCCACT |  |
| GAPDH-F | TGACTTCAACAGCGACCCA | RT-qPCR for reference gene |
| GAPDH-R | CACCCTGTTGCTGTAGCCAAA |  |
| ALDOA-15b-F | GTGCCGCGCGGCAGCCATATGCCGTACCAGTATCCGGCTCTGACT | ALDOA recombinant expression |
| ALDOA-15b-R | GTTAGCAGCCGGATCCTCGAGTTAGTACGCGTGGTTGGACACGAA |  |
